# Is it time for mental health services to invest in neurostimulation? An economic evaluation of transcranial magnetic stimulation therapies for the treatment of moderate to severe treatment-resistant depression in the UK

**DOI:** 10.1136/bmjment-2025-302237

**Published:** 2026-01-27

**Authors:** Edward Cox, Jing Ma, Cristina Roadevin, Richard Morriss, Marilyn James

**Affiliations:** 1 Nottingham Clinical Trials Unit, University of Nottingham School of Medicine, Nottingham, UK; 2 Academic Unit of Mental Health and Neuroscience, University of Nottingham School of Medicine, Nottingham, UK

**Keywords:** Mental Health Services, Depressive Disorder, Social Sciences, Mental Health

## Abstract

**Background:**

Although transcranial magnetic stimulation (TMS) protocols are safe and efficacious therapies for treatment-resistant depression (TRD), they remain inaccessible for many people in the UK and internationally. One of the main reasons for this is a lack of evidence demonstrating their value-for-money to commissioners.

**Objective:**

To assess the cost-effectiveness of repetitive transcranial magnetic stimulation therapy (rTMS) and intermittent theta-burst stimulation (iTBS) versus treatment-as-usual (TAU) for treating TRD in UK mental health care services, and to evaluate operational circumstances underpinning cost-effectiveness.

**Methods:**

This economic evaluation used data from the BRIGHTMIND trial (n=255), the SMD trial (n=187) and a study-specific structured expert elicitation exercise (n=7) to model the cost and consequences for each alternative. All findings were produced on a probabilistic basis from a Markov model using Monte Carlo simulation methods. Cost-effectiveness was assessed via incremental cost-effectiveness ratios (ICERs) per quality-adjusted life-year (QALY) gained over an 18-month time horizon from the perspectives of the UK’s NHS and personal social services and from a broader societal perspective recognisant of informal care hours and productivity costs. Scenario analyses and an operational sensitivity analysis explored the impacts alternative methodologies, service delivery cases and perspectives had on base case findings.

**Findings:**

From a health service perspective, rTMS and iTBS had pairwise ICERs of £12 093 and £12 959 per QALY compared to TAU, respectively. When incrementally compared, iTBS had an ICER of £16 621 versus rTMS. From a broader societal perspective, both rTMS and iTBS reduced informal care hours and were cost-saving compared with TAU. Study findings were particularly sensitive to service delivery, with the probability of being cost-effective ranging from 98% with high throughput and prompt delivery to approximately 4% with low throughput and prolonged delivery.

**Conclusions:**

TMS therapies improve health, reduce informal care requirements, reduce health service utilisation and offset their costs when considered in terms of productivity losses to society. rTMS and iTBS are cost-effective and should be considered for wider clinical implementation provided they are delivered at sufficient scale and in a time-efficient manner.

**Clinical implications:**

TMS can serve as a cost-effective alternative for treating moderate to severe depression after second-line treatment failure with non-psychological therapies.

**WHAT IS ALREADY KNOWN ON THIS TOPIC**

- Major depressive disorders are one of the leading causes of disability worldwide and one of the fastest-growing contributors to the global burden of disease.
- Numerous studies have demonstrated transcranial magnetic stimulation therapies (TMS) are safe and efficacious treatments for treatment-resistant depression, but their adoption in publicly funded mental healthcare services is extremely limited in the UK and globally.
- TMS has been shown to be cost-effective in various international settings, but evidence is limited in the UK, and it remains unclear which delivery protocols, if any, represent a good investment for health services and society.

**WHAT THIS STUDY ADDS**

- TMS therapies are likely to be cost-effective for adults with a history of non-response to two or more non-psychological treatments and cost saving when productivity costs are considered.
- Cost-effectiveness is highly contingent on implementation; delivery models within the limited number of practices currently offering TMS treatment in the UK suggest TMS is cost-effective.

**HOW THIS STUDY MIGHT AFFECT RESEARCH, PRACTICE OR POLICY**

- Study findings provide original evidence to policymakers and commissioners that can directly contribute to implementation strategy and provide a rationale for informed decision-making on how limited resources are allocated.
- Further clinical and economic evidence on TMS design, delivery and longer term effectiveness is needed.

## Background

 The Global Burden of Disease study (2024) places depressive disorders as the second highest disease burden worldwide,^1^ and according to the WHO, is projected to rank first by 2030.^2^ The burden of major depressive disorders (MDDs) is most pronounced in people with treatment-resistant depression (TRD), a refractory form of depression in which patients have limited or no improvement with conventional treatments, incurring higher treatment costs and poorer health-related quality of life (HRQoL) compared with non-TRD MDD.^3 4^

Transcranial magnetic stimulation (TMS) therapies are increasingly recognised as a treatment option for TRD, designed to administer magnetic pulses to stimulate cortical regions implicated in depression.^5^ Prior research has shown repetitive transcranial magnetic stimulation (rTMS) to be safe and efficacious compared with pharmacotherapy and sham treatments for TRD.^6^ More recent developments in intermittent theta-burst stimulation (iTBS) and personalisation using MRI and neuro-navigation have shown further promise in alleviating depressive symptoms.^7 8^ To deliver rTMS or iTBS, service providers require trained staff and a dedicated treatment space equipped with a stimulator device, coil and adjustable treatment chair. Protocols vary; however, treatment typically consists of 20–30 neurostimulation sessions on an outpatient basis over a four-to-six week period.

In 2015, the National Institute for Health and Care Excellence (NICE) approved TMS for the treatment of depression.^9^ Despite this and other international recommendations for TMS,^5 7 10^ implementation has been variable in the UK’s NHS and internationally, with access typically restricted only to those patients who can access and afford treatment privately.^11^ One of the main reasons for hesitancy among public healthcare commissioners is the lack of evidence demonstrating the cost-effectiveness of TMS provision within mental healthcare services.^12^ The design, delivery protocols and the evidence base for TMS have developed considerably in recent times, yet there still exist no UK economic evaluations of TMS therapies, or international evidence as to the operational circumstances under which TMS is likely to be cost-effective to health services and wider society. To address this, we present a de novo economic evaluation of TMS therapies rTMS and iTBS within the setting of UK mental healthcare services.

### Objective

To establish the value for money rTMS and iTBS can provide from a health service and wider societal perspective compared with treatment-as-usual (TAU) in TRD and to evaluate the operational circumstances underpinning cost-effectiveness.

## Methods

### Patient population

The analysis considered TMS-naïve adults diagnosed with TRD, defined by a history of non-response to two or more non-psychological treatments, experiencing moderate to severe depression at presentation in accordance with the Hamilton Depression Rating Scale (HDRS).

### Evidence

In the absence of contemporary direct comparative evidence between TMS therapies and TAU in the UK, a decision-analytic model (DAM) was developed to integrate evidence from three primary data sources: the BRIGHTMIND trial,^8^ the Specialist Mood Disorder (SMD) trial^13^ and findings from an expert elicitation exercise.

The BRIGHTMIND trial was a five-centre, parallel, double-blind, randomised controlled trial (RCT) that tested the efficacy of neuro-navigated MRI iTBS in reducing depression symptoms measured by the 17-item HDRS rating scale at 8, 16 and 26 weeks compared with rTMS delivered at a standard stimulation site in patients with TRD.^8^ Participants with moderate to severe TRD were randomly assigned to 20 sessions of either iTBS (n = 128) or rTMS (n = 127). Marked and persistent decreases in observed and self-rated measures of depressive symptoms were seen over 26 weeks in both arms.

The SMD trial was the first UK multicentre outpatient RCT in patients with persistent moderate or severe unipolar depression in the UK.^13^ The trial compared collaborative care involving combined psychological and pharmacological treatment delivered by a specialist mental health depression service (SDS) (n=93) with usual specialist mental healthcare under the direction of a consultant psychiatrist (n=94). The study found decreases in HDRS assessment scores from baseline to 6, 12 and 18 months in both arms, with statistically significant differences in improvement observed at 18 months for SDS. In this setting, TAU for TRD consists of stepped and matched care models comprising antidepressant or antipsychotic medications, outpatient cognitive behavioural therapy or problem-solving treatments, and occupational or peer support. More intensive community or inpatient therapies (eg, electroconvulsive therapy (ECT)) are offered in higher risk situations (eg, self-neglect, potential harm to others, suicide or medical complications in response to treatment). TMS, ketamine derivatives or psilocybin options are not routinely offered.^14^

A structured expert elicitation (SEE) exercise, designed and conducted specifically for this study, provided evidence for (1) the longer term efficacy of a single effective course of TMS; (2) the rate at which improvements in depression symptoms occur with TAU; and (3) the average costs of introducing TMS into routine NHS mental healthcare services. Protocolled methods and findings are described briefly here and in full in the online supplemental files 1 and 2. The SEE interviewed clinical experts highly experienced in the delivery of TMS therapies and the treatment of patients suffering from depressive disorders. The SEE was protocolised and analysed in line with Bjoke *et al*’s^15^ recommendations, including extensive piloting; experts independent from study design and conduct; pre-exercise training on study context, expressing uncertainty and relevant biases; individual-level elicitations; recording conflicts of interest; variable interval measures for expressing quantitative parameters (adapted from STEER materials, online supplemental file 1); linear pooling across experts; and exploring between expert variation.

### Decision analytical model

A multistate Markov model was used to evaluate the cost-effectiveness of delivering TMS therapies rTMS and iTBS into UK mental health services compared with TAU. The DAM has four depression-related health states defined in accordance with the HDRS categorisations reported in NICE depression guideline update GC90 (2017): Remission (0–7), Mild (8–13), Moderate (14–22) and Severe (23+) (online supplemental figure S6). During each cycle, individuals can either: (1) remain in their current health state; (2) transition to any other depression-related health state; or (3) transition into an absorbing death state (possible from all states). This represents a departure from the remission, response and relapse models typically used in TRD.^16^ This approach was chosen because it allows individual participant level data from the SMD and BRIGHTMIND trials to provide a more granular assessment of clinical trajectories of patients while avoiding the need to assign fixed and clinically meaningful thresholds for response and remission that are orthogonal, mutually exclusive and do not overly homogenise treatment response.^17^ The analysis considered rTMS and iTBS as adjuncts to TAU with consequent changes in depressive symptoms the only mechanism in which benefits from TMS could accrue in the model. To ensure a fair comparison, each alternative considered identical population characteristics and baseline depression health-state membership. The model was run over an 18-month time horizon (informed via SEE), applied a half-cycle correction, used a 2-week cycle length and discounted costs and outcomes at 3.5% per annum.^18^ A full summary of the variables used in the economic model is reported in online supplemental file 3.

### Transitions

Transitions into the death state occur irrespective of treatment or health-state membership and follow UK age-adjusted and sex-adjusted general population mortality risks elevated by the relative risks of all-cause mortality in persons with severe unipolar depression (online supplemental table S29).^19^ For TAU, transitions between depression states directly follow the trajectory observed in the usual specialist mental healthcare arm from the SMD trial. It was assumed transitions occurred uniformly between biannual assessments (online supplemental figure S5 and table S5). Since usual care in mental healthcare services has developed since the SMD trial, transitions informed via the SDS arm (a more enhanced form of care) were also considered in a scenario analysis. For iTBS and rTMS, transition matrices were derived using multistate models from BRIGHTMIND HDRS data at baseline, 8, 16 and 26 weeks (online supplemental tables S26–S8). After 6 months, it was assumed no further improvements above TAU were feasible, and that the cohort would transition to the health state composition observed in TAU in accordance with expert opinion on the longer term efficacy of effective courses of TMS. The maintenance in treatment effect was fitted using a Weibull regression (online supplemental figures S1–S4 and tables S2–S4). Due to the significant uncertainty in longer term efficacy of TMS therapies, scenario analyses considered: (1) a best-case scenario assuming all improvements achieved up to 6 months are maintained up to 18 months; (2) a worst-case scenario in which improvements only extend up to 6 months post-initiation; (3) estimates specifically from the most optimistic expert; and (4) estimates specifically from the most pessimistic expert.

### Costs

The costs for patients in the model were estimated from an NHS and personal social services (PSS) perspective (‘health-service*’* hereafter). A broader societal perspective recognisant of productivity costs and informal care hours was also evaluated.

Intervention costs were derived from the resource use requirements stated by experts, equipment costs provided by the manufacturer of the TMS equipment used in the BRIGHTMIND trial, and staff time, training and imaging (iTBS) unit costs from PSSRU and NHS reference costs, respectively (online supplemental tables S6–S18). In line with the BRIGHTMIND trial, it was assumed that iTBS would be targeted using a functional and resting state MRI scan. Equipment costs were annuitised over the 10-year lifetime of the machine and divided by average reported throughput (ie, number of patients treated per year per machine). Equipment costs were inclusive of the machine as well as a replacement coil and leads, an extended warranty and two product services. A scenario analysis considered intervention costs aligned to TMS delivery observed in the BRIGHTMIND trial.

Health state costs were estimated by regressing observed health-service costs from the SMD trial onto health state membership using generalised estimating equations controlling for age, sex, time and site effects (online supplemental table S25).^13^ Values were then inflated to the study costing year (2022/2023). SMD findings were selected in the base case analysis because the trials prolonged and regular follow-up intervals were conducive to panel regression methods. A scenario analysis considered using cross-sectional generalised linear regression with BRIGHTMIND costs accrued over 16 weeks and health state membership defined at 8 and 16 weeks (online supplemental table S24).

Health state productivity costs were calculated using a human capital approach. Self-reported work days lost to depression in the BRIGHTMIND trial were costed using the UK’s Office for National Statistics employment-family linked earnings and stratified by health state membership at 8-week and 16-week observations (0–16 week definition). A scenario analysis considered costs and health state membership observed at 16–26 weeks.

### Outcome measures

In this cost-utility analysis, the primary outcome of interest was quality-adjusted life-years (QALYs). QALYs are a composite measure of health in which benefits, in terms of length of life, are adjusted to reflect quality of life (utility), with one QALY equal to 1 year of life in perfect health. Health state utilities were estimated using an ordinary least squares panel regression approach with dependent cross-walked EQ-5D-3L scores and baseline utility, health state, age, sex, ethnicity, time and site effects as regressors (online supplemental table S19–S23). BRIGHTMIND findings were selected in the base case analysis due to a larger sample, greater health state dispersion and closer alignment to findings from the literature. SMD trial findings using the same approach were considered in scenario analysis.

Since informal care does not have a market price, unpaid hours dedicated by friends or family members were considered as a secondary outcome within the broader societal perspective.^20^ Health state values were calculated like productivity costs from average responses in the BRIGHTMIND trial at 0–16 weeks and 16–26 weeks in a scenario analysis.

### Analysis

Cost-effectiveness was evaluated by incrementally comparing the mean cost and QALY estimates for each alternative, alongside the corresponding incremental cost-effectiveness ratios (ICERs) to provide the additional cost of an intervention per QALY gained. In line with NICE recommendations, ICERs were appraised against the threshold range of £20 000–£30 000 per QALY.^18^ To aid interpretation, base case findings are presented in separate rTMS and iTBS pairwise comparisons with TAU and then jointly in a fully incremental assessment.

Non-linearities between model inputs and outputs meant all base case, scenario and sensitivity analyses were conducted on a probabilistic basis using Monte Carlo simulation (concurrently sampling from the distributions of input parameters, see online supplemental table S20). Each probabilistic analysis comprised 5000 simulations with mean convergence evaluated. Cost-effectiveness acceptability curves (CEAC) were used to illustrate decision uncertainty at willingness-to-pay thresholds (λ) up to £100 000 per QALY gained. Scenario analyses considered: (1) alternative longer-term maintenances; (2) no mortality; (3) SMD trial population and model parameters; (4) BRIGHTMIND trial population and model parameters; (5) SDS definition for TAU depressive symptom trajectory; (6) BRIGHTMIND trial TMS delivery costs; and (7) alternative 16–26 week calculation of informal care and productivity costs. Operational sensitivity analysis explored how individual and simultaneous changes in annual TMS equipment throughput, the number of sessions delivered per course and the average time taken to deliver a TMS session impacted the distribution of simulated health-service costs and the consequent changes in cost-effectiveness for rTMS and iTBS versus TAU. In the absence of a robust dose–response relationship, treatment efficacy was assumed independent of the time/number of sessions within the plausible ranges defined by the SEE (online supplemental file 2).

## Findings

The sociodemographic and clinical characteristics of patients from the BRIGHTMIND and SMD trials, and those used to define the population within the DAM, are published elsewhere^8 13^ and are summarised in online supplemental table S21. Depression-related clinical characteristics were largely comparable between studies, consistent with the use of analogous inclusion/exclusion criteria. Demographic characteristics differed in that participants from the SMD study were recruited from less geographically dispersed centres, more typically female, and were less likely to be in full-time employment.

### Structured expert elicitation

From seven participating clinical experts, all provided evidence on operational matters, five provided responses on time to improvement with TAU and four stated their beliefs on the longer term efficacy of an effective course of TMS compared with TAU (online supplemental table S1). Experts were in consensus that, in the absence of boosters, benefits were largely consigned to ≤18 months besides a small number of exceptional responders. The general trajectory of maintenance among those benefitting from TMS was a more robust short-term impact, followed by an intermediary drop-off in maintenance between 6 and 12 months, culminating in a gradual decline up to 18 months. Experts’ perception of time to improvement with TAU did not conflict with uniformity, as they deemed improvements were highly variable in practice. Experts cited few concerns with site TMS equipment, technical support, training or administration in their practices. From expert responses, we derived an average course of treatment comprising approximately 27 30-minute sessions using a machine treating 43 patients a year (base case composition of TMS care). A full summary of findings from the SEE is reported in online supplemental file 2.

### Cost-effectiveness

Intervention costs (rTMS: £1184; iTBS: £1440) to the health service were partially offset by wider savings in healthcare utilisation (rTMS: £603; iTBS: £670). From the health-service perspective, rTMS and iTBS had pairwise ICERs of £12 093 and £12 959 per QALY gained compared with TAU, respectively, indicating they would both represent a cost-effective investment in care compared with TAU (table 1). When incrementally compared, iTBS had an ICER of £16 621 versus rTMS.

**Table 1:** Base case incremental cost-effectiveness findings

Health service perspective					
	Costs	QALYS	Incremental		
			Costs	QALYs	ICER
rTMS vs TAU					
TAU	£5940	0.9253			
rTMS	£6521	0.9734	£581	0.0480	£12 093
iTBS vs TAU					
TAU	£5940	0.9253			
iTBS	£6709	0.9847	£770	0.0594	£12 959
Incremental comparison					
TAU	£5940	0.9253			
rTMS	£6521	0.9734	£581	0.0480	£12 093
iTBS	£6709	0.9847	£189	0.0113	£16 621

Broader societal perspective							
	Costs	QALYs	Informal care hours	Incremental			
				Costs	Informal care hours	QALYs	ICER
rTMS vs TAU							
rTMS	£9861	0.9734	217				
TAU	£9880	0.9253	240	£19	28.4	−0.0480	Dominated
iTBS vs TAU							
iTBS	£9864	0.9847	211				
TAU	£9880	0.9253	240	£16	28.4	−0.0594	Dominated
Incremental comparison							
rTMS	£9861	0.9734	217				
iTBS	£9864	0.9847	211	£3	−5.7	0.0113	£269
TAU	£9880	0.9253	240	£16	28.4	−0.0594	Dominated

ICER, incremental cost-effectiveness ratio; iTBS, intermittent theta-burst stimulation; QALYs, quality-adjusted life-years; rTMS, repetitive transcranial magnetic stimulation therapy; TAU, treatment-as-usual.

For the broader societal perspective, both rTMS and iTBS dominated TAU, being more effective and associated with lower costs that, on average, reduce informal care hours by over 28 and 34 hours compared with TAU, respectively. When incrementally compared, iTBS had an ICER of £269 versus rTMS. Intervention costs, including the additional imaging costs necessitated by guided iTBS, were largely offset when considering saving to both productivity (rTMS: £600; iTBS: £786) and wider healthcare utilisation.

**Figure 1:**
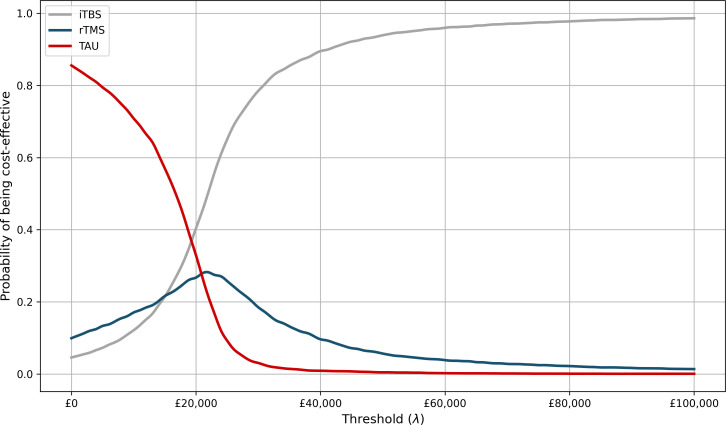
Cost-effectiveness acceptability curves. iTBS,intermittent theta-burst stimulation; rTMS, repetitive transcranial magnetic stimulation therapy; TAU, treatment-as-usual.

Figure 1 displays the CEAC illustrating the level of decision uncertainty that exists across varying levels of willingness-to-pay. Across NICE’s threshold range, both rTMS and iTBS were more likely to be cost-effective than TAU from a health service perspective. Beyond £20 000/QALY, iTBS was increasingly the most likely cost-effective alternative. Below £20 000/QALY, TAU typically had the highest likelihood.

**Figure 2:**
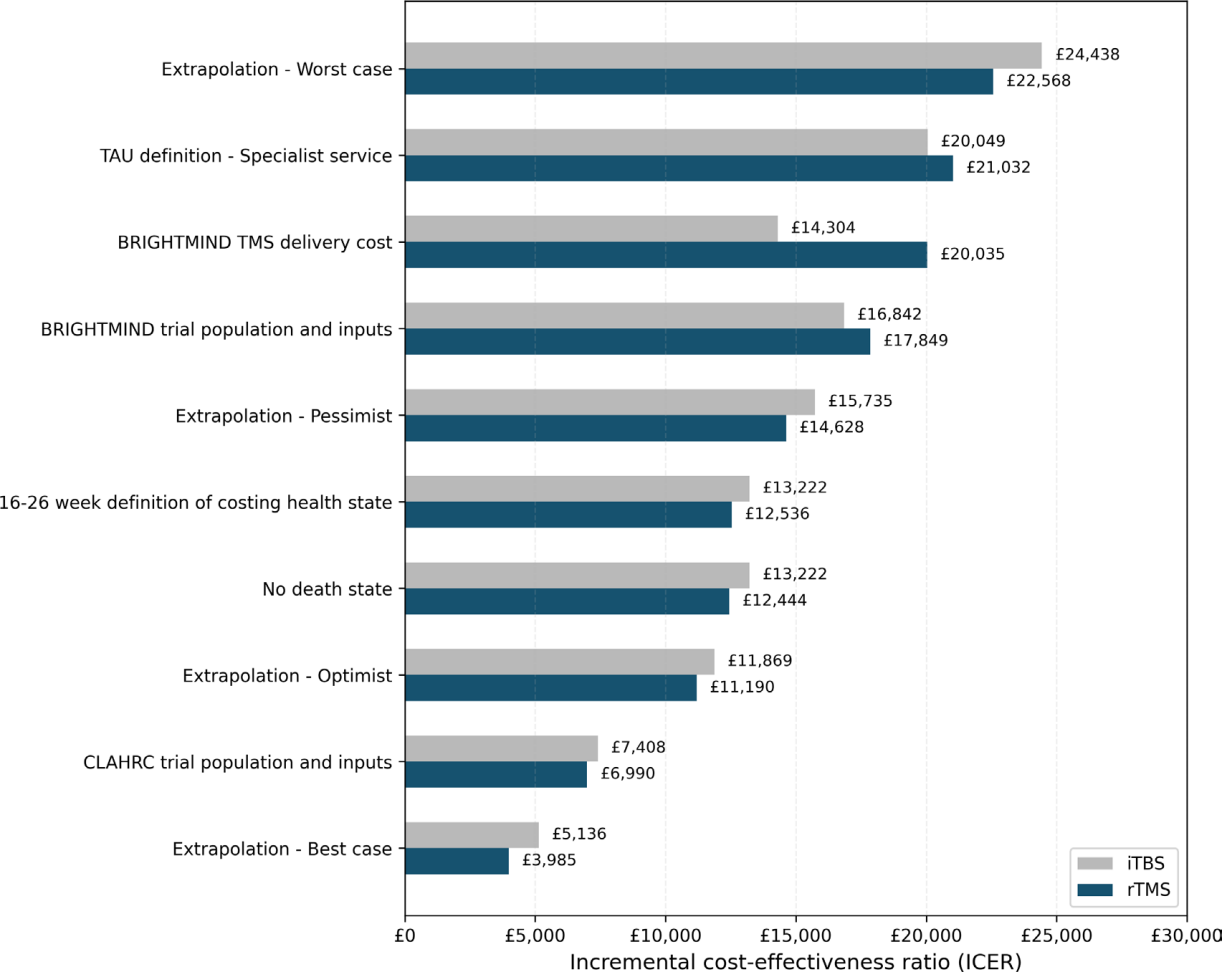
Scenario analyses pairwise incremental cost-effectiveness ratios (ICERs). iTBS, intermittent theta-burst stimulation; rTMS, repetitive transcranial magnetic stimulation therapy; TAU, treatment-as-usual.

Figure 2 reports the ICER estimates for a range of scenario analyses. The results generally support the base case conclusions on cost-effectiveness, with no scenarios having an ICER exceeding NICE’s maximum £30 000/QALY threshold. The majority were below £20 000/QALY. ICERs only fell within the NICE threshold range when assuming the worst-case scenario for the extrapolation of TMS maintenance, an SMD definition of TAU efficacy, and when applying BRIGHTMIND TMS delivery costs. For each scenario analysis, see online supplemental table S30 for fully incremental results and online supplemental figures S7–S16 for health state membership plots.

**Figure 3:**
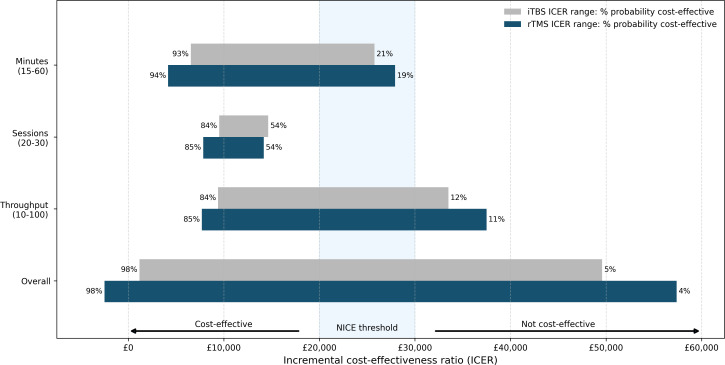
Operational sensitivity analysis. ICER, incremental cost-effectiveness ratio; iTBS, intermittent theta-burst stimulation; rTMS, repetitive transcranial magnetic stimulation therapy; % probability cost-effective, likelihood of intervention being cost-effective versus treatment-as-usual at minimum and maximum bounds when λ=£20,000.

The operational sensitivity analysis found study findings were particularly sensitive to the time required to deliver each TMS session and the throughput realised by each TMS machine (figure 3). ICERs remained below £30 000/QALY across all ranges of minutes and number of sessions considered, but exceeded this threshold when throughput fell below approximately 13 patients per year. The overall cost-effectiveness of TMS therapies appears to be highly contingent on delivery in practice, with the probability of being cost-effective ranging from 98% with high throughput and prompt delivery to approximately 4% with low throughput and prolonged delivery (online supplemental table S31).

## Discussion

Our findings suggest that rTMS and iTBS confer significant health benefits in the treatment of moderate to severe TRD and are cost-effective relative to TAU from both health service and broader societal perspectives. Findings were robust across numerous scenario analyses and decision uncertainty was low when approaching the maximum willingness-to-pay threshold of £30 000/QALY. At base case settings, rTMS and iTBS improve health outcomes while reducing wider healthcare utilisation, informal care requirements on friends and families, and productivity costs to wider society. From a health service perspective, rTMS and iTBS represent an investment in care. Initial up-front costs in equipment and configuration culminate in downstream improvements in mental health outcomes and savings to health services compared with TAU. These costs are entirely recovered when also considering societal productivity savings.

No previous UK studies have evaluated the cost-effectiveness of rTMS and MRI and neuromodulation guided iTBS for the treatment of TRD compared with usual specialist mental healthcare under the direction of a consultant psychiatrist. Furthermore, previous studies have not explored in detail the perspectives and specific conditions under which TMS is likely to represent good value for money. Study conclusions on cost-effectiveness from a health service perspective align with recent international studies finding positive QALY gains (≤0.1), additional health service costs for TMS compared with TAU, and iTBS being more cost-effective than rTMS.^21^,^25^ It is notable that alignment exists given prior evaluations consider different populations, time horizons, international contexts, model designs and efficacy estimates than those considered here. Earlier studies found rTMS to be cost-saving from a health service perspective for populations with fewer failures with antidepressant treatment than reported here.^26 27^

Strengths of this study include data analysed from two large, contemporary and comparable UK multicentre RCTs which achieved high levels of data completion with validated HRQoL instruments and service use questionnaires. Gaps in the evidence base, including the longer term efficacy of TMS and its operational delivery, were informed via leading experts interviewed within a study-specific protocolled SEE. The decision analytical model structure provided more granular and defined stratification of depression status than typical ‘response’ and ‘remission’ models, and study methods align to NICE guidance for economic evaluations in health technology assessment.^18^ Health state cost and utilities from BRIGHTMIND and SMD trials were externally valid, used in tandem and separately in base case and scenario analyses, and controlled for relevant covariables to address potential baseline imbalances. The impacts of alternative methodologies, service delivery cases and perspectives on base case findings were also explored.

Nonetheless, the analysis had limitations. The primary limitation concerns the absence of direct comparative evidence between TMS therapies and usual care for TRD in UK mental healthcare services. Consequently, this study relies on an indirect comparison between TMS in BRIGHTMIND and TAU in the SMD trial. Although differences in baseline characteristics and depression status were modest and adjusted for in the DAM and its parameterisation, the approach incurs a higher risk of bias than if patient clinical trajectories were informed via a direct comparison within an RCT achieving covariate balance. In addition, the study’s broader societal perspective was limited in scope. Impacts from improvements in depression on education, welfare benefits, social care requirements and private out-of-pocket payments were not measured, meaning societal savings from TMS likely exceed those reported here. Conversely, the analysis did not capture the considerable burden frequent attendances for TMS sessions pose to patients. The magnitude of this burden will vary considerably according to contextual (eg, geographical access) and operational (eg, same-day sessions) factors. Other limitations include the protocol target sample in the SEE (n=5) was not achieved for questions regarding longer term efficacy (n=4), the impacts of structural uncertainty on findings are unknown besides the removal of the death state, and there exist several limitations to external generalisability. These concern the atypical TMS schedule adopted in BRIGHTMIND (20 protocolled 60-minute appointments, rather than typical protocols of 20–30 TMS sessions of shorter duration), the utilisation of MRI scans and neuronavigation to guide iTBS, something largely not used in the UK or internationally due to capacity constraints, and results are specific to TAU being defined within UK specialist mental healthcare services. In other settings, usual care might be delivered in non-specialist settings (eg, primary care) or incorporate ketamine derivatives, electroconvulsive therapy (ECT) or vagal nerve stimulation.^10^ Although both trials included adults below the age of 25 years and over 65 years, the majority of the sample were between these age groups. Some caution should be applied when extrapolating to younger populations when medication may be less effective and older populations in whom ECT is used more often.

### Clinical implications

TMS has been recommended clinically for sequential use after second-line treatment failure with pharmacotherapies and psychological therapies, before combined pharmacological and psychological treatments or ECT because of superior tolerability.^28 29^ Such an approach may also serve as a cost-effective intermediary step for treating moderate to severe depression,^13 30^ requiring less specialist expertise and incurring less risk to patient safety (eg, anaesthetic or cardiometabolic risk).

Study findings suggest that the cost-effectiveness of TMS is contingent on sizeable throughput and streamlined delivery. Our SEE demonstrates that TMS delivery is flexible, and where currently available in the UK, caters to specific contextual needs and circumstances. TMS can be delivered multiple times a day (saving travel and administrative burden), most healthcare professionals can be trained to deliver it, administration protocols can be as short as 15 min, and the procedure has modest delivery requirements. Whether the conclusions here are generalisable to any specific service depends on how similar their delivery model is to the present modelled intervention. Our findings suggest that TMS delivered at scale is likely to be cost-effective. From the UK’s perspective, TMS is already approved by NICE and can be delivered into primary care, specialist or community mental health services. Such delivery aligns with objectives set out in the NHS Long Term Plan to increase labour market participation through health interventions by linking new and integrated models of mental healthcare support to adults with severe mental illnesses. Productivity and broader societal gains could be enhanced further still via the integration of TMS into pre-existing employment support programmes such as Individual Placement and Support (IPS), a programme designed to help individuals with severe mental health problems secure and sustain paid employment. The centralised delivery of TMS in settings of high need with pre-existing mental health services represents the easiest starting points for expansion. Future research should assess TMS in head-to-head comparisons vs usual care, better establish the durability of improvements from TMS on patient-centred outcomes, evaluate dose-response relationships to optimise provision and review real-world delivery.

This study provides evidence that TMS, in the form of rTMS or iTBS, is cost-effective at UK willingness-to-pay thresholds relative to TAU for the treatment of moderate to severe TRD. TMS should be considered for wider clinical implementation, provided services can achieve sufficient throughput and streamlined delivery.

## Supplementary material

10.1136/bmjment-2025-302237 online supplemental file 1

## Data Availability

Data are available upon reasonable request.
