# Supplementary material for: Is it time for mental health services to invest in neurostimulation? An economic evaluation of transcranial magnetic stimulation therapies for the treatment of moderate to severe treatment-resistant depression in the UK
Source: BMJ Ment Health. 2026 Jan 27;29(1):e302237. doi: 10.1136/bmjment-2025-302237 (PMC12853435; doi:10.1136/bmjment-2025-302237)
Supplement: online supplemental file 1 [file bmjment-29-1-s001.pdf]

Supplementary Appendix

Contents

1 Supplementary 1 - Structured Expert Elicitation Protocol 2
- 1.1 Background . . . 2
- 1.2 Rationale . . . 2
- 1.3 Protocol . . . 3
  - 1.3.1 Experts . . . 3
  - 1.3.2 Quantities elicited . . . 3
  - 1.3.3 Approach to elicitation . . . 4
  - 1.3.4 Method . . . 4
  - 1.3.5 Aggregation . . . 4
  - 1.3.6 Delivery . . . 5
  - 1.3.7 Training and piloting . . . 5
  - 1.3.8 Rationales and documentation . . . 5

2 Supplementary 2 - Structured Expert Elicitation Findings 6
- 2.1 Background . . . 6
- 2.2 Adherence to protocol . . . 6
- 2.3 Experts . . . 7
- 2.4 Materials . . . 8
- 2.5 Results . . . 11
  - 2.5.1 Question 1 - longer-term efficacy of magnetic stimulation . . . 11
  - 2.5.2 Question 2 - time to improvement . . . 14
  - 2.5.3 Question 3 - operational delivery of TMS . . . 15

3 Supplementary 3 - Decision Analytic Model Inputs and Additional Findings 23
- 3.1 Model schematic . . . 23
- 3.2 Economic model inputs . . . 23
- 3.3 Missing data from the BRIGHTMIND trial . . . 25
- 3.4 Economic model probabilistic parameters . . . 26
- 3.5 Participant characteristics . . . 27
- 3.6 Regression outputs . . . 28
- 3.7 Transition matrices . . . 29
- 3.8 Mortality risk and life tables . . . 31
- 3.9 Health-state membership . . . 31
- 3.10 Scenario analyses . . . 37
- 3.11 Operational sensitivity analysis . . . 42

1 Supplementary 1 - Structured Expert Elicitation Protocol

For the economic evaluation of magnetic stimulation therapies in the UK

January 22, 2025

1.1 Background

The BRIGHTMIND trial tested the effectiveness of treating moderate to severe treatment resistant depression (TRD) using two magnetic stimulation approaches: (i) repeated transcranial magnetic stimulation therapy (rTMS), a non-invasive treatment that uses magnetic fields to stimulate nerve cells in the brain; and (ii) connectivity-guided intermittent theta burst stimulation (iTBS), a patterned high-frequency form of rTMS that targets neural circuits using functional connectivity data.[1]

We seek to provide relevant stakeholders (including patients living with TRD, local and national commissioners, and health-care providers), with evidence of their cost-effectiveness in treating moderate to severe TRD compared to treatment as usual (TAU) in current UK mental health services.

In the absence of contemporary direct comparative evidence, we have developed a decision-analytic model (DAM) to evaluate the cost-effectiveness of each magnetic stimulation therapy approach versus TAU. The DAM incorporates evidence from the BRIGHTMIND trial, literature sources, and makes an indirect comparison to TAU using data from the SMD trial, which studied the effectiveness of a specialist depression service compared with usual specialist mental health care provision in England.[2]

The BRIGHTMIND study assessed participant outcomes up to 26 weeks and found both rTMS and iTBS demonstrated substantial improvements in observed and self-rated measures of depression, including clinically important changes in self-rated anxiety, functioning and quality of life. In both arms, around one-third of participants with moderate to severe TRD showed a response, one-fifth achieved remission and one-fifth maintained a sustained response for 6 months.[1]

The SMD trial however also demonstrated that, in the same population, those receiving TAU (which does not include magnetic stimulation therapy) also improve over time.[2] Specifically, the trial found those randomised to usual specialist mental health care services achieved continual improvements in the overall distribution of depression scores over successive 6-month follow-up assessments.

This begs the question: how do the improvements in depressive symptoms from magnetic stimulation therapy shown in BRIGHTMIND compare to those experienced within specialist mental health care services, and do they represent a cost-effective investment in care?

1.2 Rationale

At present, our ability to answer this question is limited by three gaps in the evidence base:

(1) The longer-term efficacy of a single course of magnetic stimulation therapy;
(2) The rate at which improvements in depression symptoms occur with TAU;
(3) The likely average costs of introducing magnetic stimulation therapies into routine NHS mental health care services (e.g., administration, training, maintenance, throughput, etc.,)

The BRIGHTMIND study showed the greatest reduction in depression scores at the first 8-week follow-up. Assessments at 16- and 26-weeks found these initial improvements were broadly maintained, albeit with a gradual increase in the proportion experiencing severe depression (as characteristed on the HDRS scale[3]). In SMD, participants who received usual specialist mental health care saw less marked improvements by 6 months, with fewer people in remission or experiencing a milder form of depression compared to rTMS or iTBS in BRIGHTMIND. However, outcomes with TAU continued to improve during the trial, and by 12-months a similar proportion were experiencing severe depression (20% versus 21%) than at BRIGHTMIND study end. By 18-months, the proportion of patients experiencing an improved

HDRS severity state compared to baseline (69%) was equal between SMD TAU and BRIGHTMIND participants.

With limited evidence on the longer-term benefits of a completed course of magnetic stimulation therapy,[4] and considering the complex natural histories of this population,[5] we seek to broadly understand the time-horizon over which magnetic stimulation therapies may realistically offer continued benefit to patients above TAU.

We reason there are two factors that will drive the health gains we can expect to achieve from the implementation of magnetic stimulation therapies in mental health services; (1) for patients benefitting from magnetic stimulation therapy, how long do we expect a course of treatment to provide continued benefit above routine care; and (2) what is a realistic trajectory of improvements for those receiving TAU. Given the high fixed equipment costs of rTMS and iTBS, and the significant number of appointments required to deliver a course of TMS therapy, we also believe a third factor will be crucial for establishing the cost-effectiveness of magnetic stimulation therapies: how will the therapy be delivered in routine clinical practice? This includes, but is not limited to, the acquisition of TMS machines, the number of sessions required in a course of treatment, typical session times, training, machine throughput, maintenance, and other factors. To inform these model inputs, we seek expert opinion and intend to conduct a robust structured expert elicitation (SEE) exercise.

1.3 Protocol

This protocol follows the general structure and recommendations outlined in Bjoke et al's reference protocol for the design and conduct of structured expert elicitations in health technology assessment.[6]

1.3.1 Experts

We will seek to recruit a minimum of five experts, all with experience and expertise in the delivery and effectiveness of magnetic stimulation therapies for the treatment of depression. Recruitment will be aided by Richard Morris, an expert in the field, and facilitated by Edward Cox. All experts recruited to the SEE will not have been involved in the development of the task.

To promote diversity of views and experiences, we will strive to recruit willing experts from different treatment centres across the UK. The normative skills required for answering the questions in the SEE will be reviewed at the start of each session (see Section 1.3.2). Any conflicts of interest in the wider adoption of magnetic stimulation therapies within the NHS will be recorded as the final question in the SEE. Expert responses will not be directly identifiable. Elicited values, and any conflicts of interest, will be reported individually and collectively alongside the publication of the primary economic evaluation.

1.3.2 Quantities elicited

The following quantities will be directly elicited from the experts:

- Question 1a: For how many months can patients continue to benefit from a single course of magnetic stimulation therapy compared to TAU?
- Question 1b) From those benefitting, what proportion continue to benefit from a single course of magnetic stimulation therapy compared to TAU over the specified time horizon (question 1a)?
- Question 2: For patients improving on TAU, for how many months might they have experienced observed improvements between bi-annual assessments?;
- Question 3: What are practical cost considerations worthy of acknowledgement in the delivery of magnetic stimulation therapies to patients in the NHS (e.g., equipment, training, maintenance, throughput, administration, etc.,)

For simplicity, differences in the longer-term effectiveness between rTMS and iTBS will not be differentiated (n.b., iTBS is not commonly administered). Only observable integers ('how many months') and proportion quantities ('what percent') will be elicited. These, we hope, will be interpretable quantities that impose an acceptable cognitive burden. Dependence between variables will not be explored.

Questions will be made as clear as possible to best fit with experts mental models. Specifically, we will provide a formal description of each question, and for Questions 1) and 2) (quantitative parameters), a more colloquial version

will be provided along with visual representations of what is being asked. Questions will be reviewed by at least two co-authors.

1.3.3 Approach to elicitation

To maximise participation, the SEE will be conducted on an individual basis using Microsoft Teams conferencing software. Meeting times and dates will be aligned to experts' schedules. Video calls will only be recorded if verbal permission is granted at the start of the call.

Specifically, the exercise will comprise:

1. The motivation behind the research and the SEE
2. An introduction into interpreting averages, variability, and uncertainty
3. An example question to aid usability and understandings for the exercise
4. A description of relevant cognitive and motivational biases
5. A brief review of related evidence from the BRIGHTMIND and SMD trials
6. Questions with one-to-one support and time and space made to record reflections

Information on the potential for bias, specifically cognitive and motivational factors, will be provided to experts in the instructions section of the elicitation exercise. Specifically, these details, alongwith instructions on interpreting normative values, will be taken from template slides designed by the STEER structured expert elicitation framework group.[7]

We will aim to conduct each SEE over an hour period. The SEE will follow the following agenda:

1. Self-introduction – 5 minutes
2. Exercise set-up – 5 minutes
3. Exercise background and instructions – 10 minutes
4. Question 1 – 20 minutes
5. Question 2 – 8 minutes
6. Question 3 – 10 minutes
7. Conflicts / Final wrap-up / Thank you – 2 minutes

All efforts will be made to clarify any concerns or queries to best help experts' understanding of what is being asked of them. In the event that we cannot keep to schedule, we will offer an extension to the meeting; rearranging a follow-up call at their convenience; or to complete the exercise unassisted at another time.

1.3.4 Method

All quantitively elicited values (questions 1 and 2) will use a variable importance measure (VIM) approach to elicit values. Specifically, we will use a chip and bin approach, a method which captures variability in expert judgments by allowing them to allocate weights (chips) based on their perceived likelihood of different outcomes. The resulting distribution will reflect the experts' assessment of relevant outcomes and their uncertainty surrounding their likelihood.

The VIM exercise will be adapted from the chips and bins template designed by the STEER structured expert elicitation framework group.[7] This includes the ability for experts to define the range of plausible values, to allocate chips/weights to those values, and an automated summary providing a descriptive review of their responses for verification.

1.3.5 Aggregation

Statistical distributions will be fitted to experts' individually elicited judgements. Within-expert uncertainty and between-expert variation will be explored in analysis. Following individual-level fitting, a pooled summary of the individual distributions will be obtained using linear pooling with equal weighting of experts. Internal review amongst co-authors

will be used to assess validity. Any internal disagreements regarding the validity of expert responses will be resolved using external validation.

1.3.6 Delivery

Throughout the SEE experts will receive feedback, this will include auto-generated summaries from chip and bin exercises, reflection time following each response and an optional ‘rationale for beliefs’ questions. Following feedback, throughout the exercise, and post-exercise where appropriate, experts will have the opportunity to revise their distributions.

The delivery will follow the design and schedule found in Section 1.3.2.

1.3.7 Training and piloting

Piloting will be conducted with a lay member of the pubic, a collaborating health economist, and reviewed by a collaborating clinical expert. Training of experts will include a review of the research background, brief overview of relevant findings from the BRIGHTMIND and SMD trials and information on relevant normative and cognitive bias concepts. More specific details on training can be found in Section 1.3.2.

1.3.8 Rationales and documentation

This protocol is intended to be the key source document detailing the methods employed, and their justification. Note the design and methods are most closely aligned to those recommended Bojke et al.[6] To promote transparency, the findings from the SEE and the SEE exercise document will be made available as supplementary documents alongside the publication of the primary economic analysis.

Rationales for the experts themselves will be recorded as best possible (session recordings, meeting notes, and via the ’Additional comments and rationale for beliefs’ question following quantitative questions.

2 Supplementary 2 - Structured Expert Elicitation Findings

2.1 Background

We conducted a structured expert elicitation (SEE) exercise to ascertain information on three parameters which lack clinical or operational evidence, but likely underpin the cost-effectiveness of transcranial magnetic stimulation therapies (TMS) in the UK:

1. The effective time-horizon and longer-term efficacy for a completed course of TMS compared with treatment as usual;
2. The speed at which improvements in depressive symptoms typically occur with treatment as usual (TAU);
3. The likely average costs of introducing TMS therapies into routine NHS mental health care services (e.g., administration, training, maintenance, throughput, etc.,)

2.2 Adherence to protocol

All structured expert elicitations largely followed the pre-specified protocol. Questions and response fields were reviewed and piloted by Sandy Ma (Health Economist), Emma Nicholson (lay reviewer with experience in software development), and Richard Morris (clinical expert) and revised according to their feedback. Experts were all highly experienced in the delivery of TMS therapies and each operated in a unique clinical practice across England (where TMS therapies are most commonly administered in the UK). All elicitations included pre-specified training materials that were successfully reviewed by all experts prior to answering questions (research background, exercise examples/instructions, findings from relevant research, information on normative and cognitive biases) with any queries addressed in real time. All meetings were conducted online. Questions 1 and 2 were answered using a variable importance measure method to capture expert opinions on an individual basis using adapted STEER materials.[7] All meetings were recorded to aid documentation and all results presented here align with protocol approaches to aggregation. For full transparency, the protocol for the SEE and the exercise sheet completed by the clinical experts is also available for review (see supplementary materials). All deviations from the protocol are included below:

Deviations:

- VIM scaling: Due to issues with macro permissions on NHS devices, and the inability to use Macros with Excel Online, for a number of experts (n=2) the range of values in the VIM exercises were kept within their maximum and minimum bounds (0-100%), rather than those tailored to expert stated bounds (see STEER template[7]). This pragmatic decision led to a reduced level of precision in our elicited estimates from these experts.
- Sample size: Our intended sample size was for 5 experts to complete all questions. This was mostly achieved. In total, 7 clinical experts contributed to the SEE. Two experts jointly provided answers to question 3 (operational questions surrounding the delivery of TMS treatment), but did not feel capable of answering questions 1 and 2 (longer-term efficacy, time to improvement between TAU assessments). Four experts completed all questions. One expert answered questions 2 and 3 but could not provide definitive answers to question 1.
- Reflection time: Due to time constraints, reflection time was generally below what we had anticipated. Although not directly timed, this can be indicated by typically little provided in the *"Additional comments and rationale for beliefs"* section of the document. Nevertheless, summary graphics from question 1 (summarising their overall findings from answering each question) were a very useful tool for contextualisation that prompted half of the experts (n=2/4) to go back and revise their responses.
- Misinterpretation: It became clear from the initial interviews that question 2, which asked experts *"the average time you would expect a patient reporting an improvement in symptoms to have experienced such improvements in the past 6-months?"*, was interpreted as - *"time to improvement"* (i.e., 2 months didn't represent 2 months with improvement, but rather represented the average expected time for the improvement to have transpired). Following this interpretation that independently emerged in all of the first three interviews, the question was altered to the following: *"What is the average time you would expect to wait for a patient receiving treatment as*

usual to experience an improvement reported at 6-months?". In all interviews prior to the question change, it was confirmed that this was their interpretation of the question.

2.3 Experts

Table S1 below displays a description of the experts who took part in the SEE, with a brief overview of their expertise, any declared conflict of interests and what questions they answered. All experts were clinical psychologists (bar one psychiatric nurse lead), independent of the design and conduct of the SEE and wider study, and had a variety of relevant backgrounds. Specifically, experts were highly experienced with treating patients suffering from depressive disorders, each provided care in different centres across the UK, and all are proficient in the delivery of TMS therapies in current clinical practice. Further details regarding the background and experience of the experts recruited to participate can be found in their full profiles cited in Table S1. Expert responses are pseudo-anonymised, meaning responses/results here are presented in a non-informative order, thereby not linkable to any one expert.

Table S1: Expert overview

Experts	Expertise	Conflict of interest*	Q1	Q2	Q3
Dr Alex O'Neill-Kerr	General adult psychiatrist, medical director, and clinical lead at Transforming Mind Solutions clinic. Specialist in TMS therapy [8].	No conflict declared		✓	✓
Dr Michael Kurkar	Clinical Director of mental health services in Oldham and leads the TMS service [9].	No conflict declared	✓	✓	✓
Dr Mohamed Abdelghani	Senior consultant psychiatrist who pioneered the introduction of TMS in UK clinical practice (London) [10].	No conflict declared	✓	✓	✓
Dr Mourad Wahba	Mourad Wahba is a consultant psychiatrist working in Newcastle as lead for the mood disorders research clinic (joint response) [11].	No conflict declared			✓
Ben Baxter	Clinical psychiatric nurse lead in the physical treatment centre within the CNTW NHS Foundation Trust (joint response) [12].	No conflict declared			✓
Dr Richard Barnes	Lead clinical psychologist delivering ECT and rTMS treatments within the Mersey Neuromodulation Service [13].	No conflict declared	✓	✓	✓
Dr Sudheer Lankappa	Consultant psychologist lead for the Nottingham Centre for Neuromodulation and Speciality Lead for Mental Health East Midlands [14]	No conflict declared	✓	✓	✓

*Affiliations with professional bodies, TMS organisations or private practices were not deemed as direct conflicts of interest

2.4 Materials

VIM question materials are displayed below. The full exercise is available on request.

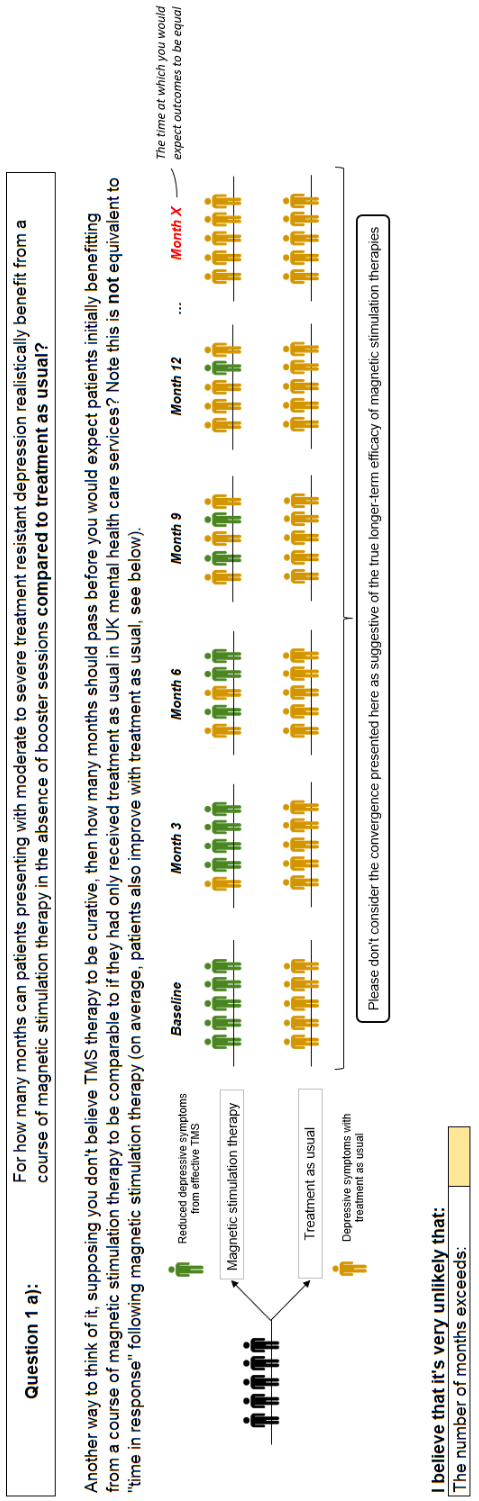

**Question 1 b):**	From those patients achieving a reduction in depressive symptoms following magnetic stimulation therapy, what proportion would you expect to continue benefiting from magnetic stimulation therapy compared to if they'd received TAU over your specified time-horizon?

Suppose we had 100 patients acheving a larger reduction in depression symptoms following a magnetic stimulation therapy compared to if they had received treatment as usual in UK mental health services. How many of these patients would you expect to continue to experience superior outcomes (without boosters) at, say, 3 months compared to if they had only received treatment as usual? 90? (90%), 70 (70%), 50 (50%)?

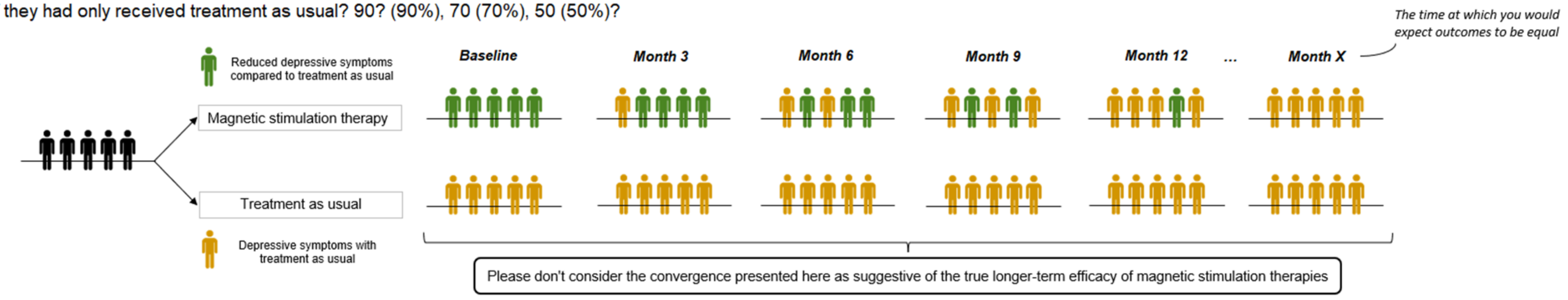

You can use the above time-marked tabs (e.g., Month 3) to populate what proportion you believe would still be benefitting with TMS (above TAU without boosters) over the time horizon you specified in Question 1a. We recommend reviewing what current evidence we have from BRIGHTMIND and CLAHRC trials below before answering:

By 3-months I believe that it's very unlikely that:

The Proportion benefiting is less than	0	%
The Proportion benefiting is greater than	100	%

Create figure

Your answer implies that there is a 98% probability that the Proportion, on average, is between 0 and 100%

Chips remaining

20

Bin	0 to 10%	10 to 20%	20 to 30%	30 to 40%	40 to 50%	50 to 60%	60 to 70%	70 to 80%	80 to 90%	90 to 100%	Total
**Number of chips**											0

Please input the number of chips you would like to place in each bin

**Question 2**	Suppose patients with moderate to severe treatment resistant depression undertake bi-annual assessments when receiving TAU from current UK mental health services. What is the average time you would expect a patient reporting an improvement in symptoms to have experienced such improvements since last follow-up?

Another way to think about it, suppose you haven't seen a patient in 6-months since their initial referral. They were diagnosed with moderate to severe treatment resistant depression. You assess the patient receiving TAU (sans TMS) now and discover they have reduced depressive symptoms. For how many months might you expect the patient to have experienced such improvements?

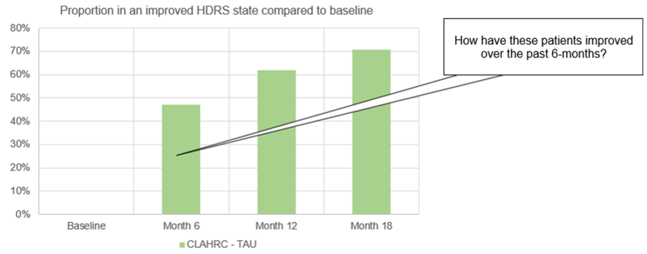

Perhaps you think in the absence of magnetic stimulation, improvements are fast to happen and are broadly maintained (green), or in fact are very slow to transpire (red), or perhaps that they are highly differentiated and a constant rate is reasonable (black)?

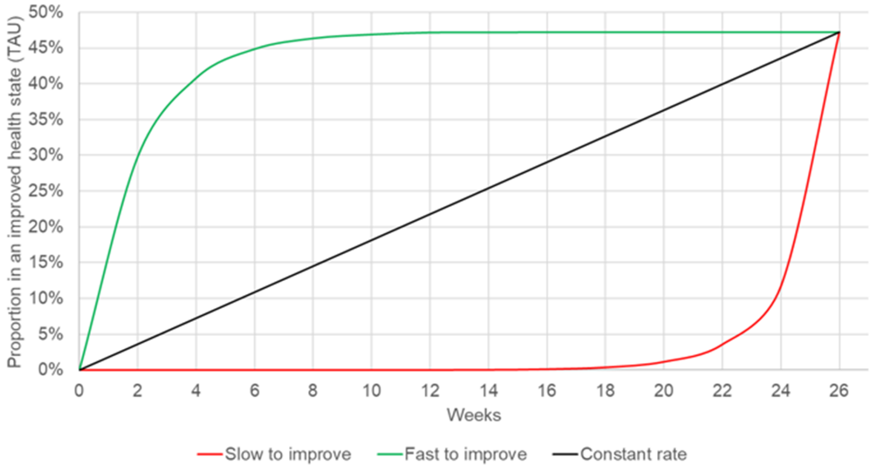

The more months people are expected to be experiencing improvements between follow-ups, the greater the proportion will be acheiving such an improvement over the 6 months

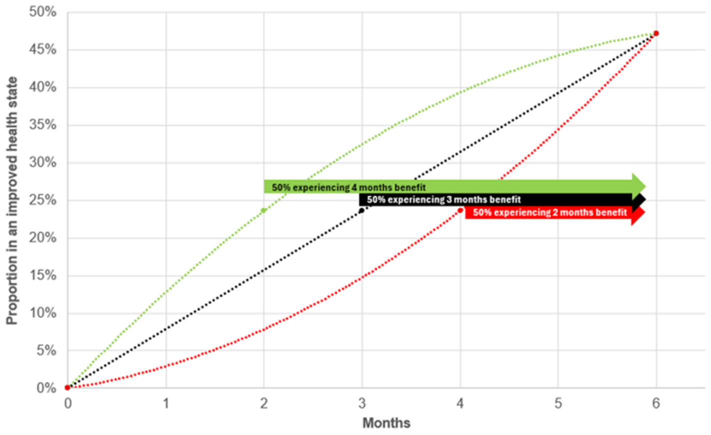

What is the average time you would expect to wait for a patient receiving treatment as usual to experience the improvement reported at 6-months?

													Chips remaining
													24
**Bin (Months)**	0 to 0.5	0.5 to 1	1 to 1.5	1.5 to 2	2 to 2.5	2.5 to 3	3 to 3.5	3.5 to 4	4 to 4.5	4.5 to 5	5 to 5.5	5.5 to 6	**Total**
**Number of chips**													0

2.5 Results

2.5.1 Question 1 - longer-term efficacy of magnetic stimulation

Question 1 a):

"For how many months can patients presenting with moderate to severe treatment resistant depression realistically benefit from a course of TMS therapy in the absence of booster sessions compared to treatment as usual?

"I believe that it's very unlikely that the number of months exceeds:"

A common clarification made by all experts for question 1a) was surrounding the interpretation of benefit. It was made clear to experts that the horizon of *"benefit"* should not be interpreted as *'time to relapse'*, but rather time until comparability in outcomes between TMS and TAU for those who stand to benefit, i.e. *'when might outcomes look similar between those benefitting from TMS and the same cohort in its absence'*. This could be via relapse, or also stem from improvements (or *'catch-up'*) from TAU. All experts regarded the time-horizon over which patients with treatment-resistant depression benefit from TMS compared to TAU as $\leq$18 months (18 months, n=3; 15 months, n=1 - see Table S2). A number of experts (n=3) believed that a small proportion of participants are likely to achieve longer-term improvements above TAU. Since question 1a) asks for a time period in which *"it's very unlikely that the number of months exceeds"*, proportions benefitting above zero at 18-months are internally consistent (see below).

Table S2: Responses to Question 1a)

Expert	Response
(1)	15 months
(2)	18 months
(3)	18 months
(4)	18 months

Question 1 b):

"From those patients achieving a reduction in depressive symptoms following TMS therapy, what proportion would you expect to continue benefiting from TMS therapy compared to if they'd received TAU over your specified time-horizon?"

Table S3 presents elicited expert opinions for the proportion of patients likely to continue benefitting from TMS compared to TAU over 18-months (see Table S2)). The aggregated distribution pooling all experts equally is also included (see protocol). The downward trajectory was broadly consistent over quarterly elicitations, albeit with measures of uncertainty between experts widely variable. This is most likely down to scaling biases: experts selecting their own ranges for the chip and bin exercise (within 0-100%) displayed markedly lower degrees of uncertainty than those utilising the entire 0-100% range. The general trajectory stated by experts was broadly consistent with a more robust short-term impact amongst those benefitting from TMS, followed by an intermediary drop-off in maintenance between 6- and 12-months, then culminating in a lower hazard rate between 12- and 18-months.

Table S3: Expert elicited answers to Question 1b

Expert	1		2		3		4		Pooled		
Summary	Mean	SD	Mean	SD	Mean	SD	Mean	SD	Mean	Between SD	Within SD
Baseline	100.00%	0.00%	100.00%	0.00%	100.00%	0.00%	100.00%	0.00%	100.00%	0.00%	0.00%
Month 3	91.50%	6.54%	88.45%	1.50%	95.00%	0.00%	85.00%	5.48%	89.99%	3.70%	3.38%
Month 6	57.08%	12.90%	70.67%	1.70%	85.00%	7.07%	60.00%	5.00%	68.19%	10.94%	6.67%
Month 9	50.00%	9.22%	55.23%	2.17%	65.00%	7.07%	35.00%	6.32%	51.31%	10.85%	6.20%
Month 12	39.50%	10.23%	49.33%	2.21%	50.00%	5.00%	9.55%	2.02%	37.09%	16.44%	4.87%
Month 15	28.00%	10.05%	31.33%	2.21%			1.00%	0.50%	20.11%	13.58%	4.25%
Month 18	12.50%	7.66%	8.55%	2.82%	24.50%	16.27%	0.00%	0.00%	11.39%	8.82%	6.69%

From these results we analysed *pooled* estimates, that being those giving an equal weighting across all expert predictions,

and also the estimates specifically from the $optimist$ (the expert with the highest values across the time-horizon (3)) and the $pessimist$ (the expert with the lowest values across the time horizon (4)) in order to provide best-case and worse-case scenarios for extrapolation.

Using only expert-elicited survival probabilities (not individual-level time-to-event data) standard methods for fitting parametric survival models are not possible. For instance, with no prior evidence of the survival probabilities, the more typical Bayesian framework using prior time-to-event data from a trial, and expert opinions from a structured elicitation exercise to jointly model survival probabilities is not possible.[15] Instead, we construct pseudo data from $pooled$, $optimist$, and $pessimist$ survival estimates that approximates the observed survival trajectory provided by the experts (n.b., where survival represents maintaining any clinical benefit above TAU). Parametric survival distributions were fitted using a weighted likelihood approach, specifically selecting models that minimise the negative log-likelihood:

$$\text{negLL}(\theta) = -\sum_i \log f\left(S_i \mid S_{\text{model}}(t_i;\theta), \sigma_i\right)$$

In the negative log-likelihood equation the summation i is taken over the specific time points at which survival probabilities were elicited. That is:

- $i \in \{0, 3, 6, 9, 12, 15, 18\}$: the quarterly time points in months used in the SEE;
- S_i: elicited survival probabilities at time i,
- σ_i: elicited standard deviations associated with S_i at time i.

At each elicited time point i, a given model $S_{\text{model}}(t_i;\theta)$ assumes that S_i arises from a normal distribution centred and dispersed around the expert predicted survival probabilities, with standard deviation σ_i. The total negative log-likelihood ($negLL$) is computed by summing the log-likelihood contributions across each of the 6 time points. For each model (S_{model}) the elicited survival probabilities S_i are treated as noisy observations assuming normally distributed errors.[16;17]

$$S_i \sim \mathcal{N}\left(S_{\text{model}}(t_i;\theta),\, \sigma_i^2\right)$$

The following parametric models were evaluated: Weibull, Gompertz, lognormal, log-logistic, exponential, gamma, and generalized gamma. Figures S1, S2, S3 present the fitted models:

Figure S1: Fitted parametric models - Pooled expert elicited answers to Question 1b

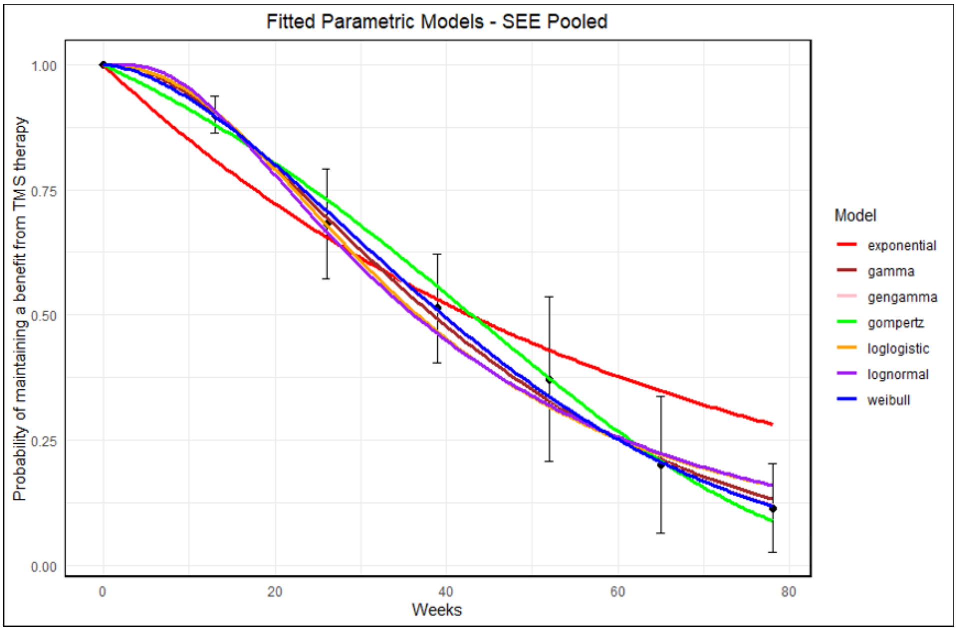

Figure S2: Fitted parametric models - Optimist expert elicited answers to Question 1b

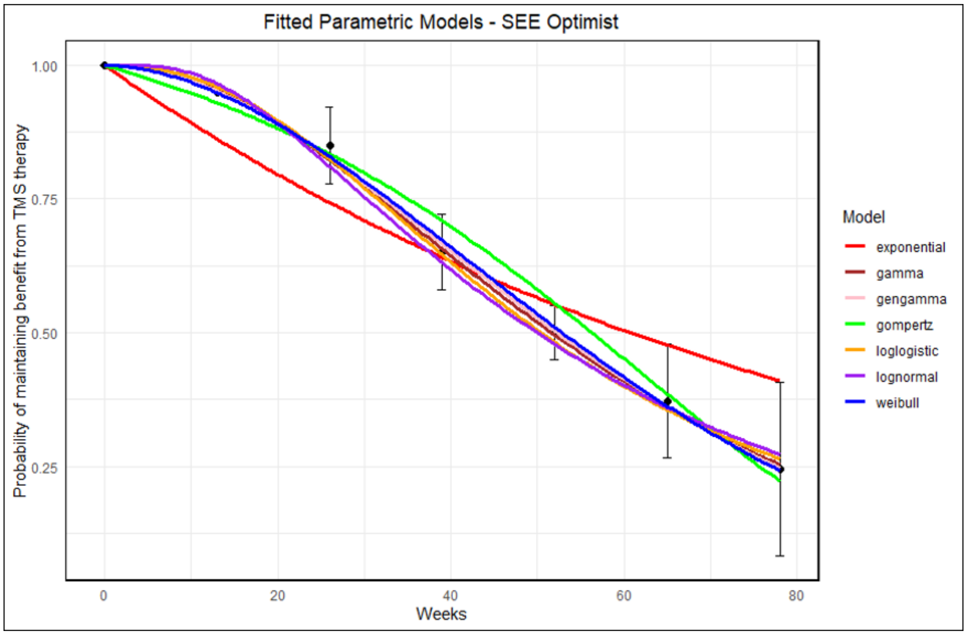

Figure S3: Fitted parametric models - Pessimist expert elicited answers to Question 1b

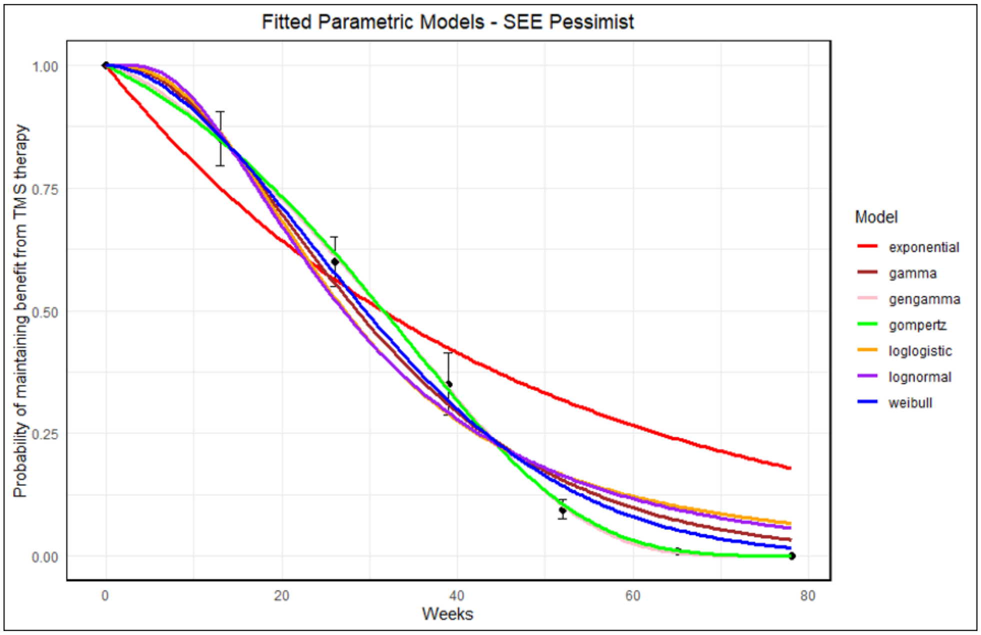

Table S4 reports the model fit statistics for each parametric model. In all cases negative log likelihood and Akaike Information Criterion were lowest using a Weibull model.

Table S4: Parametric model fit statistics

	Pooled		Optimist		Pessimist	
**Model**	**negLL**	**AIC**	**negLL**	**AIC**	**negLL**	**AIC**
weibull	-21.2871	-38.57421	-21.29828	-38.59656	-21.32098	-38.64196
gamma	-21.25866	-38.51731	-21.29404	-38.58808	-21.14867	-38.29734
loglogistic	-21.03105	-38.0621	-21.2441	-38.48821	-20.92554	-37.85107
lognormal	-20.99939	-37.99879	-21.11386	-38.22772	-20.40461	-36.80922
gompertz	-20.98467	-37.96935	-20.94844	-37.89688	-21.33219	-36.66438
gengamma	-21.2877	-36.57541	-21.30651	-36.61302	-20.29623	-36.59246
exponential	-15.86219	-29.72438	-15.93249	-29.86498	-13.06622	-24.13245

Figure S4 illustrates the selected best fitting Weibull models for each of the expert groupings considered:

Figure S4: Fitted parametric models used in the economic evaluation

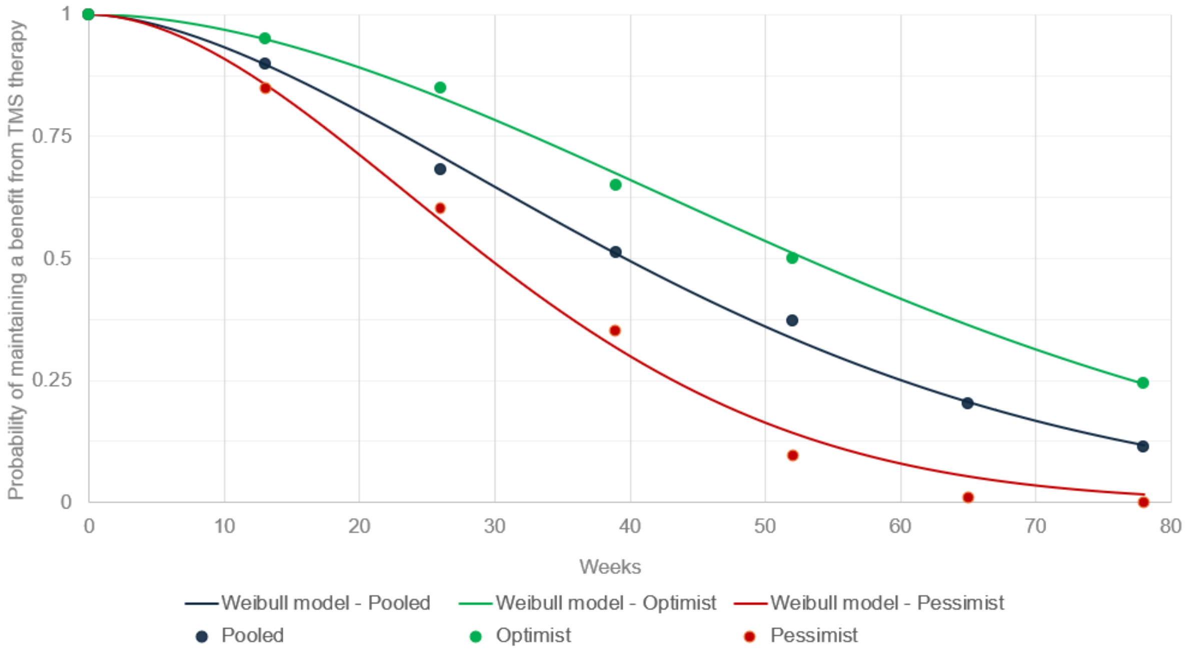

2.5.2 Question 2 - time to improvement

Suppose patients with moderate to severe treatment resistant depression undertake bi-annual assessments when receiving TAU from current UK mental health services.

"What is the average time you would expect to wait for a patient receiving treatment as usual to experience the improvement reported at 6-months?" - (n.b., see protocol deviations)

Table S5 provides the VIM distributions provided by experts, including a pooled distribution giving equal weighting to each expert response. Experts appeared to be broadly in consensus that the time, nature and extent of improvements were highly variable with TAU, and as such the **average** patient is unlikely to experience immediate improvements nor highly prolonged delays prior to their observed improvement at 6-months. Experts also highlighted that patients may have multiple marked changes in their status over this horizon. Using this evidence, we suggest that the linear interpolation of bi-annual assessments from SMD is broadly representative with expert opinion for the average population transitions between health states in TAU. Note that a uniform distribution between 0-6 months is equivalent to a linear interpolation. Patients' depressive symptoms S at month t (S_t) are equally likely to improve or deteriorate to the state ob-

served at S_{t+6} (the next bi-annual assessment), i.e., transition (T) between S_t and S_{t+6} follows: $T \sim Uniform(t, t+6)$.

Table S5: Expert elicited answers to Question 2

Bin (Months)	0 to 0.5	0.5 to 1	1 to 1.5	1.5 to 2	2 to 2.5	2.5 to 3	3 to 3.5	3.5 to 4	4 to 4.5	4.5 to 5	5 to 5.5	5.5 to 6
Expert 1	8.33%	8.33%	8.33%	8.33%	8.33%	8.33%	8.33%	8.33%	8.33%	8.33%	8.33%	8.33%
Expert 2	0.00%	4.17%	4.17%	4.17%	12.50%	16.67%	33.33%	16.67%	8.33%	0.00%	0.00%	0.00%
Expert 3	0.00%	4.17%	12.50%	33.33%	16.67%	8.33%	4.17%	4.17%	4.17%	4.17%	4.17%	4.17%
Expert 4	0.00%	0.00%	0.00%	8.33%	8.33%	8.33%	8.33%	8.33%	8.33%	12.50%	16.67%	20.83%
Expert 5	10.00%	10.00%	10.00%	10.00%	10.00%	10.00%	10.00%	10.00%	10.00%	10.00%	0.00%	0.00%
Pooled	3.67%	5.33%	7.00%	12.83%	11.17%	10.33%	12.83%	9.50%	7.83%	7.00%	5.83%	6.67%
Pooled cumulative	3.67%	9.00%	16.00%	28.83%	40.00%	50.33%	63.17%	72.67%	80.50%	87.50%	93.33%	100.00%
Linear trajectory	8.33%	16.67%	25.00%	33.33%	41.67%	50.00%	58.33%	66.67%	75.00%	83.33%	91.67%	100.00%

Figure S5 displays the cumulative distribution of the pooled expert VIM time to response at 6-months against the simple linear interpolation used in our analysis.

Figure S5: Expert elicited time to change in status observed at 6-months - answers to Question 2

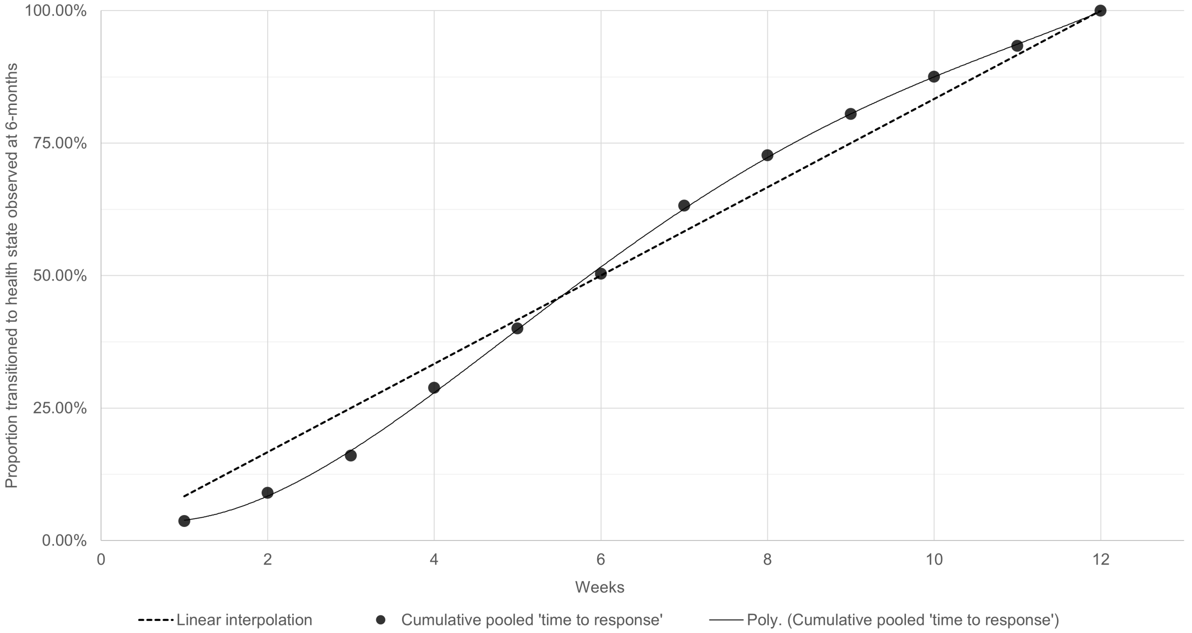

2.5.3 Question 3 - operational delivery of TMS

2.5.3.1 a) The TMS machine

What trans-magnetic stimulation (TMS) machine(s) do you use in your practice (e.g., Horizon PERFORMANCE)?

Table S6: Question 3a) responses

	Expert response	Source
(1)	MAGSTIM and MAGVENTURE machines	Self-procured
(2)	MAGSTIM Horizon Performance	BRIGHTMIND study
(3)	MAGSTIM rapid II	Self-procured
(4)	MAGSTIM Horizon with stimguide neuronavigation	Self-procured
(5)	MAGSTIM Horizon Performance	BRIGHTMIND study
(6)	MAGSTIM Horizon 3.0	Self-procured

All experts reported using MAGSTIM TMS machines (see Table S6).

Welcony, the manufacturer of MAGSTIM, has kindly provided the following costing breakdown with list prices for the latest relevant TMS MAGSTIM equipment (2025):

Magstim Horizon 3.0 StimGuide Pro (latest version of the system used in the BRIGhTMIND study): £88,500 [2-year parts and labour warranty included]

5-year warranty: £23,850

EMG/ECG electrodes (for the head tracker, 2 needed per session) 50pk: £49 EMG Lead sets 10pk (if required/not a necessity): £80

All TMS coils have a limited life. Magstim coils are 5 years/30 million pulses (if using iTBS even with moderately heavy use would cover the full five years) Replacement H 3.0 coil £9,755

Stimulators require a service at 5 and 10 years £2-2,500 approximately 10-years is the end of serviceable life.

The vast majority of equipment costs are fixed. From this we arrived at the following non-discounted ten-year fixed capital cost for the TMS equipment applicable for the delivery of TMS therapies in NHS practice:

Table S7: Question 3a) - Fixed equipment costs

**Equipment**	**Cost**
Magstim Horizon 3.0 StimGuide Pro	£88,500
Extended 5-year warranty labour/parts	£23,850
Replacement MAGSTIM coil	£9,755
Two product services	£2,500
EMG lead sets 10pk	£80
Total	£124,685

The only variable equipment cost component considered in the decision model is the £1.96 cost per session for electrodes (included within the average total cost per treatment course in the decision model). Replacement coil and EMG leads are considered as fixed expenses during the 10-year lifetime of the machine.

The annualised (A) equipment cost (C) is calculated over 10 years at discount rate r via:

$$A = C * \frac{r}{1-(1+r)^{-t}} = 124,685 * \frac{0.035}{1-(1+0.035)^{-10}} = 14,992.30$$

The annualised equipment cost per person E is calculated as the annualised equipment cost divided by the annual throughput of the machine T (i.e., number of patients treated per year per machine - see 2.5.3.4 for expert confirmation of 10-year machine horizon and for expected machine throughput over that time):

$E = A/T$ = £14992.30/43 = £348.66 per patient

Note that, as a business case, there is an exposure to repair costs between 5-10 years. However, experts all reported no or minimal repairs (inc., pedals) of their equipment, besides the costed coil replacement (see Section 2.5.3.2).

2.5.3.2 b) Maintenance

i) How much maintenance has been required from yourself/staff/department to maintain your equipment?

Experts reported maintenance requirements that largely fall within the bounds of an extended warranty and product service (e.g. faulty pedal, suspension issues). Experts reported the equipment requires *"No major upkeep"*, that maintenance is typically conducted by the manufacturer, or in one case, a third-party contractor and deemed *'effective'*. Exceptions include replacing the machine coil (costed) and one expert referring to an annual service check more regular than the manufacturer provides and that which is costed here.

Table S8: Question 3b i) responses

N	Response
(1)	"Annual service - need some repair. £6000 (to be reviewed cost) for a repair - coil is an issue."
(2)	"On average, allocate 30% of spare time for maintenance."
(3)	"Yearly checks, usually done by equipment people (nothing major) - contact magstim on one occasion)"
(4)	"Broken down after a few weeks - still in warranty. No major upkeep."
(5)	"No set standard maintenance contract- this is not recommended by the supplier. We've had issues on a couple of occasions where we had an engineer out over the past few years. One visit was just a maintenance visit, and another was to update equipment."
(6)	"routine checks, cleaning etc from PoV of staff"

ii) Specifically, have you needed any technological support, upgrades or replacement parts since receiving your TMS equipment?

Table S9: Question 3b ii) responses

N	Response
(1)	"Third party - contracted for maintenance to get any medical service. Around £10,000"
(2)	"The coil needed replacing (degrades) - needs replacing every 2-5 years. Can pick up machine's for £50,000 (theta burst needed)."
(3)	"Suspension stopped working"
(4)	"Minor replacement needed - pedal broke for raising the coil. They sent a new pedal"
(5)	" We've had issues with the coil, which happened on least three occasions- we sent this back and a replacement was sent. We incurred the charge 2/3 times. There was also a problem with the stim guide due to an error that occurred when the upgrade occurred. This was provided free of charge by the company as it was related to the upgrade."
(6)	"a number of replacement parts have been needed over the 4 years we have been using our TMS equipment. This has ben provided very efficiently by the manufacturers without charge and with minimum disruption to the service"

2.5.3.3 c) Training

What training has been necessary for your health care professionals to effectively deliver TMS therapy sessions in practice?

Experts refer to training being a mandatory outlay for the operational delivery of TMS services. While experts were not able to put an exact figure on time dedicated to the training of practitioners, it appeared at least a day or two of formal training was needed alongside observation. Many experts referred to the manufacturers helping with the delivery of training at no cost to the service.

In our analysis we have assumed that for the capacity of each machine, two practitioners will receive two full 8-hour days of training (see expert [3]), with this cost incurred twice over the course of the machine's life (every 5-years of service).

Table S10: Question 3c responses

N	Response
(1)	"Yes - training arm in private practice. Did it on the basis of ECT using those standards and competency framework. Practical and knowledge based competencies. Split to nurse competencies - 'knows about NICE guidance', practitioner needs to know it exists, a prescriber would need to have knowledge about the guidance. Morning for knowledge competencies, afternoon practical competencies. Another day come to our clinic for a week, and our practitioners will help place the coils on patients. About 10 observed placements and treatments would be signed off. I could train a practitioner and feel confident in about a week. Nurses are not used to operating machinery, can be nervous/worried. All you need is someone practical."
(2)	"We have to do a course - nurse by two consultants, support from MAGSTIM (using the machine). CPD points"
(3)	"Delivering around 2 days after training. 1 day training, another day supervising (coil, emergency stopping, cooling system fixes). Competency checklist used to demonstrate quality."
(4)	"The company provided the initial training for all staff. This was over one day. Theoretical and practical applications, both in one day. New people starting are trained by local staff now. People being trained get signed off on individual aspects (e.g. marking the cap, establishing motor threshold, procedure, treatment sites, stim guide set up) and are supervised until competent."
(5)	"A lot of training needed."
(6)	"all new staff have standard Trust induction followed by in-house theory over the use, mode of action etc., of TMS. Practical training in the use of the machines is provided by the manufacturer, with regular update training both in house and from manufacturers."

2.5.3.4 d) Throughput

i) Are you aware of, roughly, how many patients your TMS equipment has been able to deliver sessions to?

Experts reported a diverse range of throughputs at their centres (see Table S11). A common theme was that within NHS practice only 8 sessions per day per machine were realistically feasible. This would put the theoretical maximum sessions at 2920 per year per machine, equivalent to approximately 100 completed courses of treatment (assuming 29.2 sessions per patient; or 97 patients with 30 sessions). From their responses we calculated an *'on the ground'* lower average throughput as follows (expert number in parentheses - Table S11):

$$\frac{8 * 12[1] + 225/4[2] + 30[3] + 60[5] + 50/(12/18)/3[6]}{6} = 42.23$$

The throughput per year will be highly contextual given that each mental health care service will provide care for different populations under unique operational circumstances (i.e., staffing). Based on the expert opinion, the throughput per machine should fall between approximately 0 and 100 treated patients per year. The average calculable throughout from the experts was approximately 43 treated patients per year per machine. The equivalent BRIGHTMIND average site throughput (ITT) in the trial was 51. Our base case analysis will consider the aforementioned average expert throughput of 43, just below half the likely maximum capacity services can deliver. A BRIGHTMIND TMS delivery scenario will consider the average site throughput from the trial (51 treatments per year) amongst other changes, and sensitivity analysis will explore how the cost-effectiveness of TMS varies between the 10-100 range, that being the approximate bounds of the minimum expert stated throughput per machine (11) and the theoretical maximum capacity (100).

Table S11: Question 3d i) responses

N	Response
(1)	"When it closes, 120 patients on the case loads (those in treatment and on maintenance). On average, 8 patients in a chair a day, 10 would be possible but doing it. Quite intensive work, reality you could only do 8 a day. 8 patients a month by 20 treatments - running costs stay the same (staff time). Even GPs could have them."
(2)	"200-250 patients over 4 years. 180-190 at 3 -years."
(3)	"No issues with the machine for the numbers. In private can deliver 30 sessions a day on the same machine. Deliver 6-8 sessions a day in the NHS. Staffing is the main problem, very labour intense (where the main price is). In the NHS starting 9 years ago approximately 200 patients, but this is with COVID. In a year approximately 30 patients treated per machine (because of limited staff)."
(4)	"Over 100 patients."
(5)	"8-10 session per day. 60 patients in clinical service; check paper for the BRIGHTMIND numbers"
(6)	"we have had our current 3 machines for approximately 18 months and in that time have treated around 50 patients distrubuted across the 3 machines"

ii) Do you have an expected lifetime horizon for your equipment (e.g. years, number of sessions)?

Experts and the manufacturer of MAGSTIM themselves, unanimously reported the lifetime of the TMS machine to be 10-years. This is used for deriving an annuitised equipment and training cost per person over the study horizon (18 months - see 2.5.1 and 2.5.3.1).

Table S12: Question 3d ii) responses

N	Response
(1)	"Machine okay, coil will run out. They tended to use us as guinea pig, they fixed for free, maybe no longer. They will have a warranty. There aren't many major issues. They would get it sorted within a day. MAGVENTURE is Scandinavia; MAGSTIM in Wales and highly responsive."
(2)	"7-years left in its expected life time (second hand)"
(3)	"No horizon for the machine - in America the ancient TMS devices don't break easily (with coil changes). They can live longer, but the technology is moving quickly. Business case do depreciation case over 5 years - warranty runs out beyond there and you can't get refunded after 5 years."
(4)	"Expected lifespan is around 10 years from initiation."
(5)	"Trust has a policy for equipment, around 10 years."
(6)	"We would aim to renew the machines every 10 years"

2.5.3.5 e) Administration

i) How long would it typically take to deliver a session of rTMS or theta burst therapy at your practice?

Experts reported a diverse range of average administration times at their centres (see Table S13). Typically experts referenced <15 minute protocols for stimulation, but set-up and patient management can extend sessions up to anywhere between 15-60 minutes. Experts were divided on which was faster between rTMS and theta burst stimulation. Our calculated average times from expert responses were approximately 28.3 minutes for iTBS and 31.6 minutes for rTMS (expert number in parentheses):

$$iTBS: \frac{30[1] + 15[2] + 30[3] + 15[4] + 20[5] + 60[6]}{6} = 28.3$$

$$rTMS: \frac{60[1] + 15[2] + 20[3] + 30[4] + 20[5] + 45[6]}{6} = 31.7$$

Experts were not explicitly asked to outline times for iTBS, many did not have experience delivering it, and

some referenced that it *'might take longer with more targeted stimulations'* but could not provide times. Given these factors, and that calculated averages were comparable and differences by experts were largely contradictory, we decided to equalise the average session time to 30 minutes for each method (the mid-point between average times for iTBS and rTMS reported by experts). In BRIGHTMIND the average times were 52 and 56 minutes for iTBS and rTMS respectively. These will be considered in a separate scenario analysis for the BRIGHTMIND composition of care and explored across the range of values provided by experts (15-60 minutes) in sensitivity analysis.

Table S13: Question 3e i) responses

N	Response
(1)	"rTMS looking at an hour, theta burst you are looking at half-an hour. 15-20 minutes of theta burst, but with setting up and patient management closer to 30 minutes."
(2)	"15 minutes, 9 minute protocol"
(3)	"Schedule TMS session for 30 minutes, this is to review patients before the stimulation. Even if it is 3 minutes to deliver. Typically 20 minutes in and out, high frequency 19 minutes, 20 minutes for those with epilepsy. "Might take longer with iTBS for more targeted stimulations."
(4)	"rTMS is about 30 minutes, iTBS is around 15 minutes"
(5)	"15 minutes to deliver; 10 minutes in between approximately 20 minutes total per session"
(6)	"rTMS approx 45 mins, Theta burst approx 1 hour. These figures include setting up machine for patient etc., as well as time actively treating."

ii) How many sessions would you typically deliver in a treatment course?

The experts reported that a treatment course typically comprises 20 to 30 sessions (see Table S14). Experts reference 20 sessions being the original standard of care (as was the protocol in the BRIGHTMIND trial), however services now strive to deliver up to 30 sessions and potentially beyond.

Our calculated average number of sessions from expert responses were approximately 26.7 sessions (expert number in parentheses):

$$\frac{26.9[1] + 28[2] + 30[3] + 20[4] + 25[5] + 30[6]}{6} = 26.7$$

In the absence of any evidence or expert opinion to suggest differences exist between the number of sessions delivered with rTMS or iTBS, we assume an equal number (26.7). In the BRIGHTMIND trial, rTMS and iTBS averaged 19.35 and 19.13 sessions, substantially lower than what experts report is provided in routine practice. BRIGHTMIND sessions however were on average 56 and 52 minutes for rTMS and iTBS respectively, meaning the BRIGHTMIND study (primary data source for rTMS and iTBS efficacy) is misaligned with current practice by having less sessions than typically administered but with each having a longer total duration. The base case analysis assumes TMS therapy takes the form of current care, while we will explore the BRIGHTMIND composition of care as a separate scenario (one which best aligns with the source of efficacy data, but is divergent from routine care).

Table S14: Question 3e ii) responses

N	Response
(1)	"Majority fall between 20-30 session, chip and bin exercise completed (18-21 (3 chips), 22-25 (6 chips), 26-29 (5 chips), 30-33 (3 chips), 34-37 (3 chips))"
(2)	"When we started the standard was 20 sessions, now we shift towards 30 sessions (few exceptions)"
(3)	"Delivering 30 at present, although may be under dosing. In the future this will be going up, not down."
(4)	"20 sessions"
(5)	"Ideally go for 30, because of capacity issues 25 is more realistic average number of sessions"
(6)	"30."

iii) Which health care professionals typically deliver TMS therapy in your practice (e.g., title, grade, etc.,)?

Experts reported that a variety of health care professionals deliver TMS in their practice (see Table S15). The most commonly reported lead provider was a band 6 nurse. We assume for the expert defined composition of care that a grade 6 nurse will be delivering TMS (£57 per hour - PSSRU). For the BRIGHTMIND scenario analysis the average across the different health care professionals will be used (£40.83). The BRIGHTMIND average staff cost is lower on account of band 5 higher research assistants administering treatment at some sites (the remainder were band 6 nurses).

Table S15: Question 3e iii) responses

N	Response
(1)	"Health care assistant. NHS gold plate having a band 8 and band 6 working on delivery. In private practice, training for practitioners is all that is required. That's what happened with ECT. A graduate, somebody with a relevant Bsc."
(2)	"Band 7 nurse delivering - band 4-6 would be appropriate if supervised and trained"
(3)	"NHS - were nurses but occupational therapist of late (clinican with 20-25 years of experience as a care coordinator). At least masters in pschology, or clinical experience."
(4)	"Band 5 and 6 nurses"
(5)	"Band 6 nurse, three nurses. 1 nurse minimum - band 4 assistance or researcher. Ideally band 6 and 7, but not possible."
(6)	"band 6 and band 5 RMNs assisted by band 3 TMS technicians"

2.5.3.6 f) ECT

What do you see as the most significant cost factors in the delivery of electroconvulsive therapy (ECT) in NHS practice? Are you aware of any evidence for the cost of delivering ECT?

In general experts referred to electroconvulsive therapy as a very seperate form of care, one which requires hospitalisation, higher staff requirements and relatively more costly (from a health care providers perspective).

Table S16: Question 3f responses

N	Response
(1)	"£850 averaged cost per treatment - probably £1,000 per treatment. Privately £250, BUPA will pay £150. "
(2)	"Much more expensive, different patient groups (some intersection). Personale, equipment, time in hospital, side-effects, possibel cognitive impairements, long-term costs of anaesethia, more staff. At least 2-3 delivery nurses"
(3)	"Patient experience is completely different - no anasetetic. No downtime after the session, ECT do nothing for a day. No burden on care givers (no one has to accompany to the session), less invasive, less medical costs (no anaesetist or anaestistic , less staff required for delivery, less stigma, more durable effects. ECT will not maintain the benefit beyond a year - the Nottingham new survey about aceptability. Ultrasound 1st and magnetic therapies second. Outpatient procedure for rtms, hosptial inpatient delivery of ECT. "
(4)	"Most significant costs: staff time given consultant grade for anaesthetist and psychiatrist, and nursing staff with minimum band 6 and 7 nurses for ECTAS. Service agreement for equipment as well., Replacing machine would be around 30k."
(5)	"£7,800 full course of ECT; iTBS is still expensive. ECT is relatively more costly than TMS in the short-term and response is much quicker"
(6)	''Staffing and (to a lesser extent) transport costs etc incurred by patient"

2.5.3.7 g) Barriers to implementation

Are you aware of any barriers, or at least relevant considerations not mentioned thus far, in expanding TMS provision in UK mental health care services?

Table S17: Question 3g responses

N	Response
(1)	A lot of people don't know anything about it. Little neurology training in practice, like medications but little understandings around practical applications in psychology. Little procedures, surgery and the like. Younger practitioners interested though. Little belief in its effectiveness, placebo effect cited. Cost as well, NHS doesn't have the space to put them. The NHS can't expand to take on the demand. 30,000 here alone could benefit from TMS, take 30 years to get through them all. The private sector is where you can expand rapidly. Barrier around business plans for commissioners. A lot of it is ignorance, used to take quite a bit of teaching and training in the NHS, people not knowing where to get it. A number trying to get a business case and finances, how are they going to set up the service - up hill battle. It's a real shame, depression pathway and add in rTMS as part of the pathway - look at the data again and we could roll that out nationwide.
(2)	"Public awareness, professional training, initial investment, psychistry in general not used to using machines, pain specialists would use this far more than psychologists are. Lack of regulations can allow anyone to do TMS (snake oil salesmen emerge - bring reputational damage). Indirect barrier is that TMS is a very forgiving technology, difficult to cause harm. So can be used wrongly without treating the disorder but doing a disservice to the patients and the field as a whole. Not in a specific place in the treatment algorthm in NICE guidelines. TMS is an investment - benefits come 2-3 years. Hospital capacity."
(3)	"Commissioners resisted recommending it. Lack of understanding of TMS, including consultant psychiatrists. NICE vs FDA - FDA not approving"
(4)	"Mainly the cost. Staff training and awareness. Lack of clarity on its place in the treatment algorithm."
(5)	"Trust is not funding! A treatment option which is not being provided to patients. Really sub-optimal to not provide this care. Trust don't have money for it, that's the end."
(6)	"lack of commitment by trusts to neuromodulation treatments. Lack of knowledge/enthusiasm from clinicians preventing referrals"

3 Supplementary 3 - Decision Analytic Model Inputs and Additional Findings

3.1 Model schematic

The DAM has four depression-related health states (see below) and an absorbing death state (not presented) possible from all depression states.

Figure S6: Study DAM model schematic

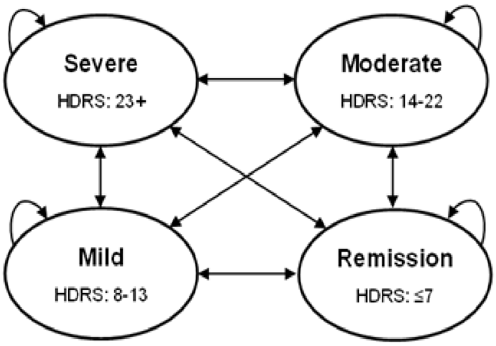

3.2 Economic model inputs

Table S18: Base case and scenario parameter values

	Base case	Source	Scenario	Source
**Baseline characteristics**				
Female	51.8%	BRIGHTMIND trial	64.0%	SMD trial
Age (years)	43.8		46.5	
Baseline health state membership				
Remission	0%	SMD trial	0%	BRIGHTMIND trial
Mild	0%		0%	
Moderate	31.3%		31.6%	
Severe	68.7%		68.4%	
**Model settings**				
Costing discount rate	3.5%	NICE guidelines		
Outcomes discount rate	3.5%	NICE guidelines		
Cycle length (weeks)	2	-		
Time horizon (months)	18	Expert opinion*		
**Costs**				
Intervention costs				
Equipment (total)	£124,685	Manufacturer	£54,000	BRIGHTMIND trial
Throughput (patients p.a.)	43	Expert opinion	102	BRIGHTMIND trial
Equipment (per patient)	£400.99	Derived*	£529.41	Derived*
Staff cost (per patient)				
rTMS	£760.95	Expert opinion	£676.93	BRIGHTMIND trial
iTBS	£760.95	Expert opinion	£729.01	
Training (total)	£1,824.00	Expert opinion	0	BRIGHTMIND trial
Training (per patient)	£21.21	Expert opinion	0	BRIGHTMIND trial
Number of sessions				
rTMS	26.7	Expert opinion	19.13	BRIGHTMIND trial

Continued on next page

Table continued from previous page

	Base case	Source	Scenario	Source
iTBS	26.7	Expert opinion	19.35	BRIGHTMIND trial
Time per session (minutes)				
rTMS	30	Expert opinion	56	BRIGHTMIND trial
iTBS	30	Expert opinion	52	BRIGHTMIND trial
MRI and fMRI scans				
rTMS	0	Expert opinion	1.08	BRIGHTMIND trial
iTBS	1	Expert opinion	1.08	BRIGHTMIND trial
Health state costs (per cycle)				
0-6 months				
Remission	£46.58	SMD trial	£31.78	BRIGHTMIND trial
Mild	£75.37		£43.54	
Moderate	£90.84		£80.75	
Severe	£150.15		£99.03	
6-12 months				
Remission	£54.27	SMD trial	£31.78	BRIGHTMIND trial
Mild	£87.81		£43.54	
Moderate	£105.83		£80.75	
Severe	£174.93		£99.03	
12-18 months				
Additional health state costs				
Remission	£34.60	SMD trial	£31.78	BRIGHTMIND trial
Mild	£55.99		£43.54	
Moderate	£67.48		£80.75	
Severe	£111.54		£99.03	
**Health state utilities**				
Remission	0.84	BRIGHTMIND trial	0.81	SMD trial
Mild	0.75		0.68	
Moderate	0.62		0.42	
Severe	0.50		0.17	
**Informal care hours**				
Remission	1.10	BRIGHTMIND (0-16 weeks)	0.42	BRIGHTMIND (16-26 weeks)
Mild	4.54		1.53	
Moderate	6.66		4.11	
Severe	7.79		8.61	
**Productivity costs**				
Remission	£19.81	BRIGHTMIND (0-16 weeks)	£0.00	BRIGHTMIND (16-26 weeks)
Mild	£21.73		£9.98	
Moderate	£112.16		£9.13	
Severe	£154.97		£134.02	
**Mortality**				
Relative risk - women	2.32		1.00	No death state
Relative risk - men	1.93	Laursen et al (2016)	1.00	

3.3 Missing data from the BRIGHTMIND trial

Table S19: Observed and missing data by variable for the BRIGHTMIND dataset

Variable	BRIGHTMIND		
	Observed	Missing	% missing
Treatment group	255	0	0%
Site	255	0	0%
Baseline severe depression (HDRS)	255	0	0%
Ethnicity	255	0	0%
Female	255	0	0%
Age	255	0	0%
Baseline inpatient costs	255	0	0%
Inpatient costs week 16	221	34	13%
Inpatient costs week 26	205	50	20%
Baseline outpatient costs	255	0	0%
Outpatient costs week 16	221	34	13%
Outpatient costs week 26	205	50	20%
Baseline community costs	255	0	0%
Community costs week 16	221	34	13%
Community costs week 26	205	50	20%
Baseline primary care costs	255	0	0%
Primary care costs week 16	221	34	13%
Primary care costs week 26	205	50	20%
Mental health medication costs week 16	215	40	16%
Mental health medication costs week 26	202	53	21%
Baseline productivity costs	254	1	0%
Productivity costs week 16	220	35	14%
Productivity costs week 26	205	50	20%
Baseline EQ-5D	255	0	0%
EQ-5D week 8	220	35	14%
EQ-5D week 16	218	37	15%
EQ-5D week 26	201	54	21%
Baseline informal care days	254	0	0%
Informal care hours week 16	219	34	13%
Informal care hours week 26	198	50	20%
Baseline informal care days off work*	254	0	0%
Informal care days off work week 16*	219	34	13%
Informal care days off work week 26*	198	50	20%

3.4 Economic model probabilistic parameters

Table S20: Specification of all probabilistic parameters

Parameter	Distribution	Mean	SD	α	B
**Health state utilities**					
Remission	Multivariate normality within an OLS regression	See Table 22			
Mild					
Moderate					
Severe					
**Health state costs**					
Remission	Multivariate normality within a GEE gamma log family link framework	See Table 25			
Mild					
Moderate					
Severe					
Productivity					
0-16 weeks					
Remission	Gamma	£17.84		18.33	0.97
Mild	Gamma	£19.57		27.62	0.71
Moderate	Gamma	£101.00		220.74	0.46
Severe	Gamma	£139.55		157.63	0.89
16-26 weeks (scenario)					
Remission	Gamma	£0.00		-	-
Mild	Gamma	£8.99		13.77	0.65
Moderate	Gamma	£8.23		13.22	0.62
Severe	Gamma	£120.69		160.77	0.75
**Informal care hours**					
0-16 weeks					
Remission	Gamma	1.0992		2.41	0.46
Mild	Gamma	4.5441		12.22	0.37
Moderate	Gamma	6.6645		13.51	0.49
Severe	Gamma	7.7857		18.12	0.43
16-26 weeks (scenario)					
Remission	Gamma	0.4205		0.62	0.68
Mild	Gamma	1.5323		2.70	0.57
Moderate	Gamma	4.1149		10.48	0.39
Severe	Gamma	8.6125		17.45	0.49
**Mortality**					
Relative risk of mortality compared to the general population					
Male	Normal	2.32	0.010		
Female	Normal	1.93	0.008		
**Transition matrices**					
iTBS	Dirichlet	See Table S8–S10			
rTMS					
TAU					

3.5 Participant characteristics

Table S21: Comparison of BRIGHTMIND and SMD trial participant characteristics (baseline)

	BRIGHTMIND		SMD	
	rTMS ($n = 127$)	iTBS ($n = 128$)	Usual care ($n = 94$)	SDS ($n = 93$)
**Demographic**				
Age (years)	44	44	46	47
Sex (female, %)	62 (48.8%)	70 (54.7%)	53 (57.0%)	60 (64.5%)
**Site**				
London	30 (23.6%)	29 (22.7%)		
Newcastle	23 (18.1%)	24 (18.8%)		
Northampton	14 (11.0%)	15 (11.7%)		
Oldham	4 (3.2%)	3 (2.3%)		
Nottingham	56 (44.1%)	57 (44.5%)	137 (73%)	
Cambridge			29 (16%)	
Derby			21 (11%)	
**Employment status**				
Full-time	38 (30.2%)	37 (28.9%)	22 (24%)	17 (19%)
Other employment	36 (28.6%)	26 (20.3%)	11 (12%)	10 (11%)
Retired	13 (10.3%)	17 (13.3%)	10 (11%)	16 (18%)
Unemployed	39 (31.0%)	48 (37.5%)	37 (41%)	36 (40%)
Receipt of benefits (Yes, n (%))	52 (40.9%)	45 (35.2%)	63 (70%)	61 (67%)
**Household characteristics**				
Marital status: married/cohabiting (Yes, n (%))	76 (59.8%)	55 (43.0%)	50 (53%)	42 (45%)
Dependants (children/other) (Yes, n (%))	42 (33.1%)	36 (28.1%)	58 (62%)	61 (66%)
**Clinical**				
Baseline BDI-I	34.4	32.3	35.6	35.9
Baseline PHQ-9 score	20.2	19.4	19.3	19.9
Baseline EQ-5D-3L index score	0.395	0.421	0.337	0.361
Years since first diagnosis of depression	6.1 years (median)		5.7	7.3
Baseline HDRS	23.9	22.9	23.2	22.0
**Inclusion criteria**				
BRIGHTMIND	HDRS17 score ≥ 16; Massachusetts General Hospital Treatment Resistant Depression staging score ≥ 2; aged ≥ 18 years; met criteria for DSM-V major depressive disorder using a structured clinical interview; had the capacity to provide informed consent			
SMD	Referrer defined primary unipolar depression; aged ≥ 18 years; received direct and continuous care from health professional(s) in preceding 6 months; under the care of a secondary care mental health team; DSM-IV (SCID) diagnosis of major depressive disorder with current major depressive episode; met five of nine NICE criteria for symptoms of moderate depression; HDRS17 ≥ 16; GAF ≤ 60; able and willing to give oral and written informed consent to participate in the study.			

3.6 Regression outputs

All regression analyses from the BRIGHTMIND and SMD trials utilised data imputed using multiple imputation using chained equations (MICE), employed to address missing data in EQ5D, costs and the HDRS scores used to define health state across all assessment time points. The imputation was implemented in Stata 18 using predictive mean matching with a single nearest neighbour, an approach that replaces missing values with observed values from cases exhibiting similar predicted means. This semi-parametric method preserves the distribution and plausibility of the imputed data while accounting for uncertainty due to missingness. The imputation models included age, sex, and study centre as auxillary variables. Twenty multiply imputed datasets were generated (aligned to the degrees of missingness) with a fixed random seed to aid reproducibility.

Health state preferences scores were estimated using OLS panel regressions with random effects.

Generalised estimating equations (GEE) models with a gamma family and log link were used to estimate health state costs. The model included health state, month, age, sex, and study centre as covariates, with an independent working correlation structure and robust standard errors to account for repeated measures and potential misspecification of the correlation structure. This approach provided population-averaged estimates suitable for the positively skewed cost data.

Table S22: BRIGHTMIND OLS EQ5D preference score regression analysis (base case)

	coeff.	standard error
Mild Depression	-0.0897793	0.0185182
Moderate Depression	-0.2169377	0.0179742
Severe Depression	-0.3431808	0.0177438
rTMS	-0.0143859	0.0180886
age	-0.000187	0.0006348
female	-0.0039173	0.0174977
Site		
Newcastle	-0.0790451	0.0291897
Northampton	0.0160181	0.0330231
Nottingham	-0.0275825	0.0232388
Oldham	-0.1220458	0.060057
Ethnicity dummy		
BAME	0.0014913	0.0323033
Constant	0.885229	0.0390134

Table S23: SMD OLS EQ5D preference score regression analysis (scenario)

	coeff.	standard error
Mild Depression	-0.134574	0.0620517
Moderate Depression	-0.4039659	0.0589312
Severe Depression	-0.6731147	0.0689367
month 6	0.0207799	0.0442186
month 12	-0.0949781	0.0410224
month 18	-0.0832991	0.0537667
age	-0.0040565	0.0023778
female	0.0680287	0.0602554
Site:		
Derby	-0.0378255	0.1249217
Cambridge	0.0040174	0.0774964
Constant	1.006726	0.1135056

Table S24: BRIGHTMIND GLM log link cost regression (scenario) *

	coeff.	standard error
Mild Depression	0.3148453	0.3863051
Moderate Depression	0.93254	0.348899
Severe Depression	1.136591	0.3926531
rTMS	0.258786	0.2519987
age	-0.0024393	0.0098132
female	0.0200204	0.2533671
Site		
Newcastle	0.294903	0.4074459
Northampton	-0.79931	0.4509688
Nottingham	-0.2743394	0.3351538
Oldham	0.0244894	0.949145
Ethnicity dummy		
BAME	-0.0965616	0.3933412
Constant	5.578609	0.5258016

*Health state membership defined by weeks 8 and 16 in BRIGHMIND

Table S25: SMD generalised estimating equation log link cost regression (base case)

**Total 18 month cost**	**Coeff.**	**Standard error**
Mild Depression	0.4812668	0.3166269
Moderate Depression	0.6678774	0.2580884
Severe Depression	1.170453	0.2933127
month 12	0.1527307	0.1551334
month 18	-0.2972739	0.199942
age	0.0012055	0.0119472
female	0.3834422	0.2412292
Site		
Derby	-0.3870313	0.2685621
Cambridge	-0.0272937	0.3331576
Constant	5.951325	0.6529824

3.7 Transition matrices

Table S26: Transition matrices - iTBS

From/*to*	*Remission*	*Mild*	*Moderate*	*Severe*
iTBS – 0–8 weeks				
Remission	1	0	0	0
Mild	0	1	0	0
Moderate	0.077580103	0.154380424	0.738251914	0.029787558
Severe	0.024300531	0.064271706	0.237838563	0.673589199
iTBS – 8–16 weeks				
Remission	0.877114374	0.089650142	0.029577109	0.003658376
Mild	0.062441891	0.877198741	0.056938092	0.003421276
Moderate	0.004564794	0.064307257	0.831036841	0.100091108
Severe	0.022612376	0.004406868	0.086237824	0.886742932
iTBS – 16–26 weeks				

From/*to*	*Remission*	*Mild*	*Moderate*	*Severe*
Remission	0.875063574	0.091293608	0.031741208	0.00190161
Mild	0.063839495	0.876563991	0.055769847	0.003826667
Moderate	0.004767199	0.063958733	0.828344142	0.102929926
Severe	0.02247201	0.005628115	0.087002736	0.884897139

Table S27: Transition matrices - rTMS

From/*to*	*Remission*	*Mild*	*Moderate*	*Severe*
rTMS – 0–8 weeks				
Remission	1	0	0	0
Mild	0	1	0	0
Moderate	0.067998772	0.13273822	0.769632052	0.029630955
Severe	0.02209218	0.020919523	0.249412043	0.707576253
rTMS – 8–16 weeks				
Remission	0.912239502	0.077026851	0.010362389	0.000371258
Mild	0.063248494	0.796312059	0.134277442	0.006162005
Moderate	0.003670115	0.093559831	0.8271457	0.075624355
Severe	0.000167063	0.006454257	0.113971925	0.879406754
rTMS – 16–26 weeks				
Remission	0.915926335	0.070886176	0.01282091	0.00036658
Mild	0.054287484	0.676342573	0.257933631	0.011436311
Moderate	0.005514687	0.142845312	0.783300026	0.068339974
Severe	0.003521747	0.096202335	0.017309851	0.882966066

Table S28: Transition matrices - TAU

From/*to*	*Remission*	*Mild*	*Moderate*	*Severe*
TAU – 0–6 months				
Remission	1	0	0	0
Mild	0	1	0	0
Moderate	0.008060095	0.021065573	0.966087272	0.004787061
Severe	0.006458127	0.000784215	0.032668251	0.960089407
TAU – 6–12 months				
Remission	0.944360965	0.010012908	0.0454253	0.000200827
Mild	0.020716916	0.89899374	0.079932675	0.000356669
Moderate	0.006538581	0.038022387	0.947049232	0.0083898
Severe	0.004031411	0.000999161	0.048423577	0.946545851
TAU – 12–18 months				
Remission	0.944360965	0.010012908	0.0454253	0.000200827
Mild	0.020716916	0.89899374	0.079932675	0.000356669
Moderate	0.006538581	0.038022387	0.947049232	0.0083898
Severe	0.004031411	0.000999161	0.048423577	0.946545851

3.8 Mortality risk and life tables

Table S29: Relative depressive mortality risks and absolute population mortality risks

Mortality	Live value	Mean	SE	Source
Mortality relative risk of depression - Male	2.32	2.32	0.010	Laursen et al (2016)
Mortality relative risk of depression - Female	1.93	1.93	0.008	Laursen et al (2016)
**Age**	2-weekly ACM risk	males	females	
43	0.00010	0.002004	0.001164	National Life Tables, England, period expectation of life, based on data for the years 2021-2023
44	0.00011	0.002164	0.001317	
45	0.00012	0.002380	0.001398	
46	0.00013	0.002514	0.001566	
47	0.00015	0.002826	0.001806	
48	0.00016	0.003080	0.001895	

3.9 Health-state membership

Health-state membership for base case and all scenario analyses are displayed on a deterministic basis.

Figures S7–S16: Modelled health-state membership by arm and scenario

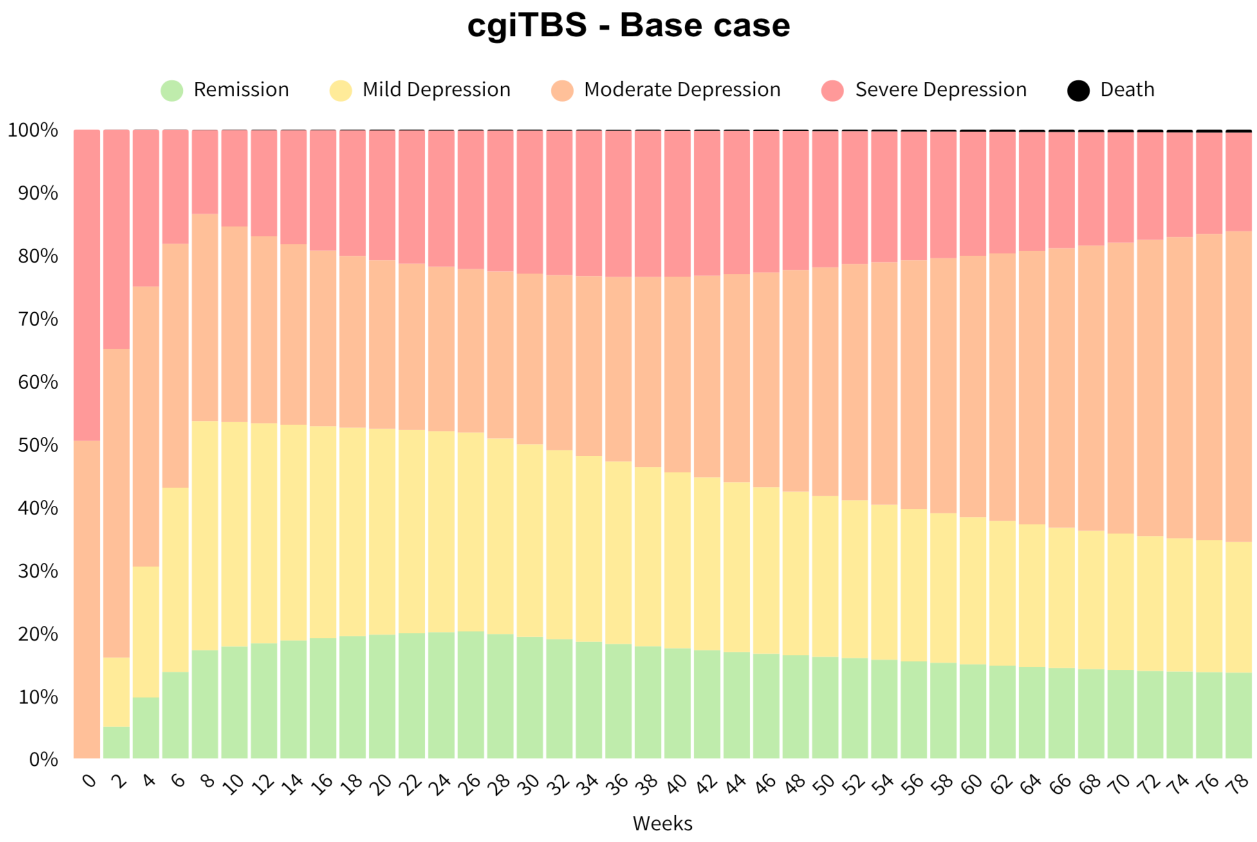

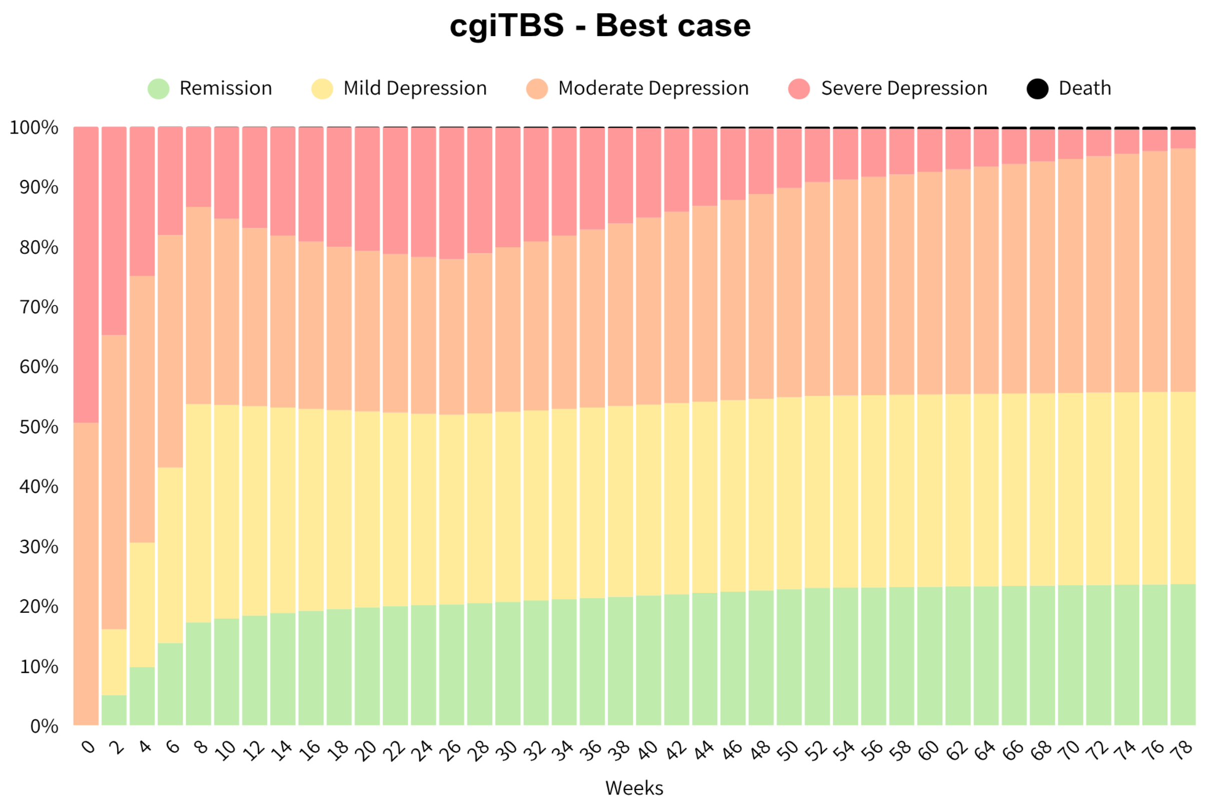
cgiTBS - Best case
Remission
Mild Depression
Moderate Depression
Severe Depression
Death
100%
90%
80%
70%
60%
50%
40%
30%
20%
10%
0%
0 2 4 6 8 10 12 14 16 18 20 22 24 26 28 30 32 34 36 38 40 42 44 46 48 50 52 54 56 58 60 62 64 66 68 70 72 74 76 78
Weeks

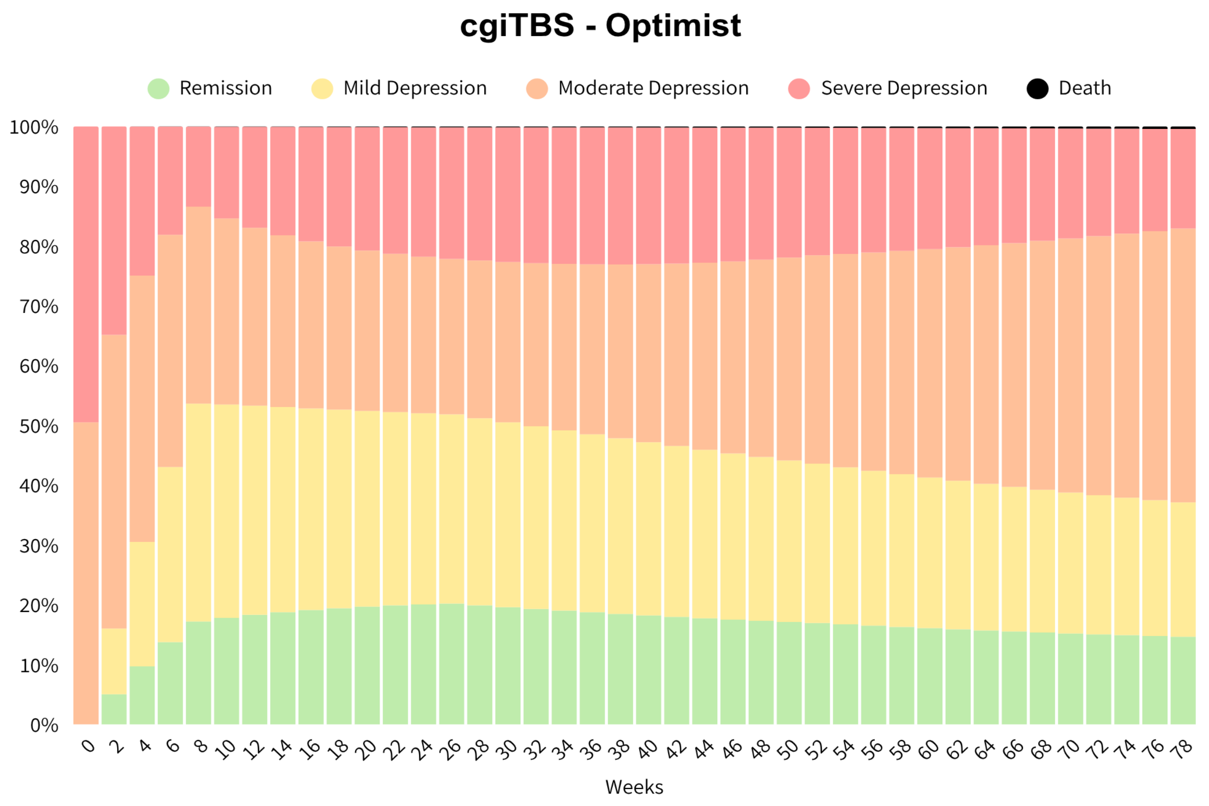
cgiTBS - Optimist
Remission
Mild Depression
Moderate Depression
Severe Depression
Death
100%
90%
80%
70%
60%
50%
40%
30%
20%
10%
0%
0 2 4 6 8 10 12 14 16 18 20 22 24 26 28 30 32 34 36 38 40 42 44 46 48 50 52 54 56 58 60 62 64 66 68 70 72 74 76 78
Weeks

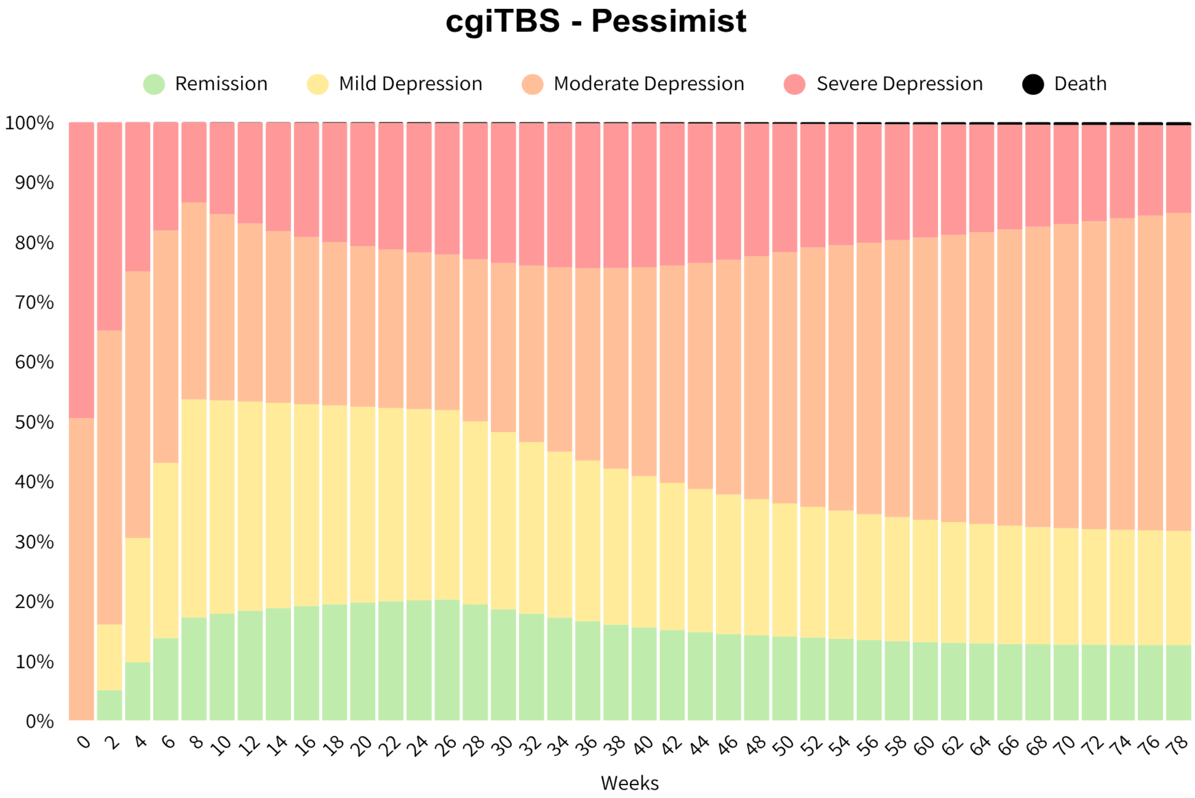
cgiTBS - Pessimist
Remission
Mild Depression
Moderate Depression
Severe Depression
Death
100%
90%
80%
70%
60%
50%
40%
30%
20%
10%
0%
0 2 4 6 8 10 12 14 16 18 20 22 24 26 28 30 32 34 36 38 40 42 44 46 48 50 52 54 56 58 60 62 64 66 68 70 72 74 76 78
Weeks

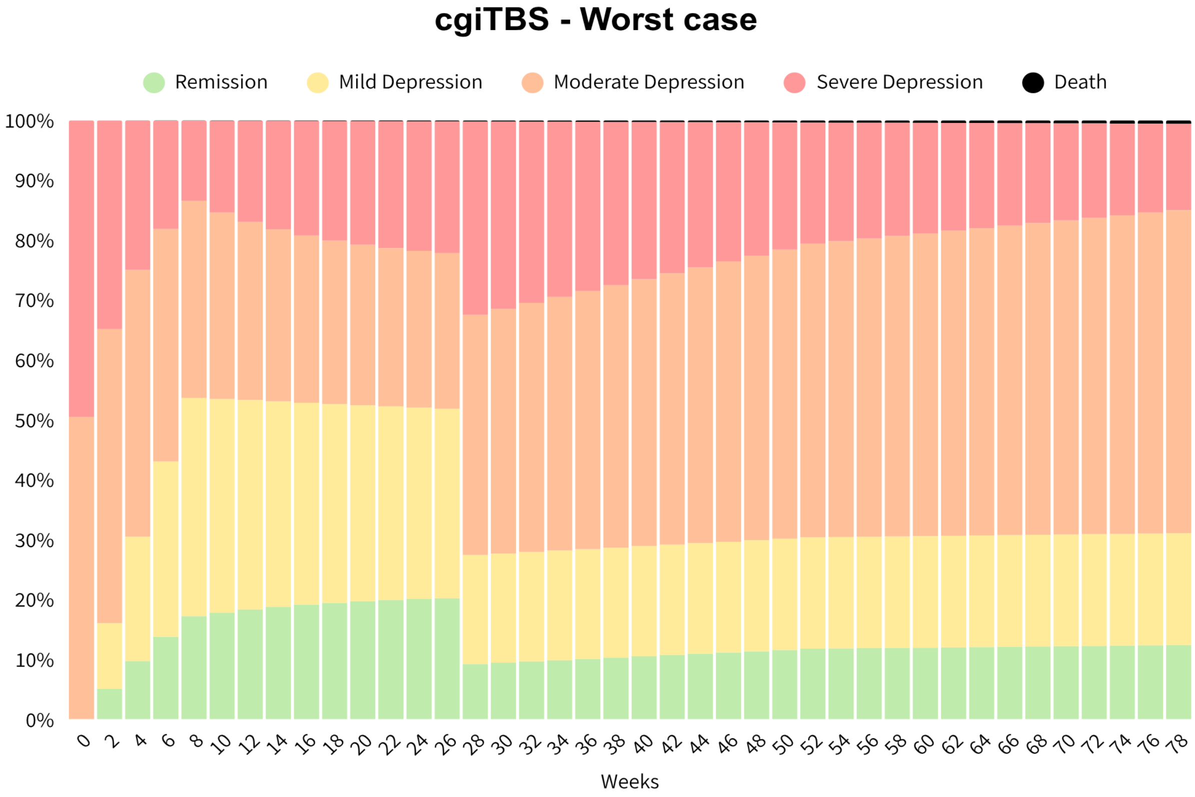
cgiTBS - Worst case
Remission
Mild Depression
Moderate Depression
Severe Depression
Death
100%
90%
80%
70%
60%
50%
40%
30%
20%
10%
0%
0 2 4 6 8 10 12 14 16 18 20 22 24 26 28 30 32 34 36 38 40 42 44 46 48 50 52 54 56 58 60 62 64 66 68 70 72 74 76 78
Weeks

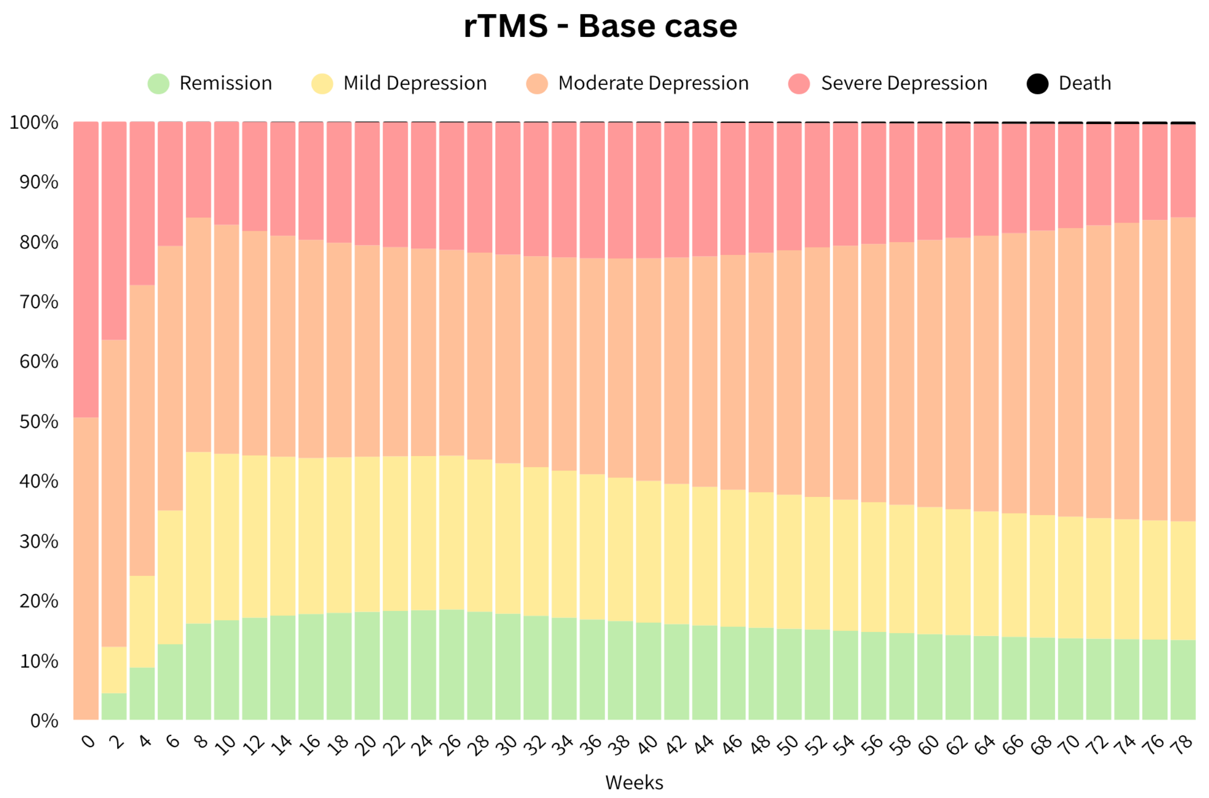
rTMS - Base case
Remission
Mild Depression
Moderate Depression
Severe Depression
Death
100%
90%
80%
70%
60%
50%
40%
30%
20%
10%
0%
0 2 4 6 8 10 12 14 16 18 20 22 24 26 28 30 32 34 36 38 40 42 44 46 48 50 52 54 56 58 60 62 64 66 68 70 72 74 76 78
Weeks

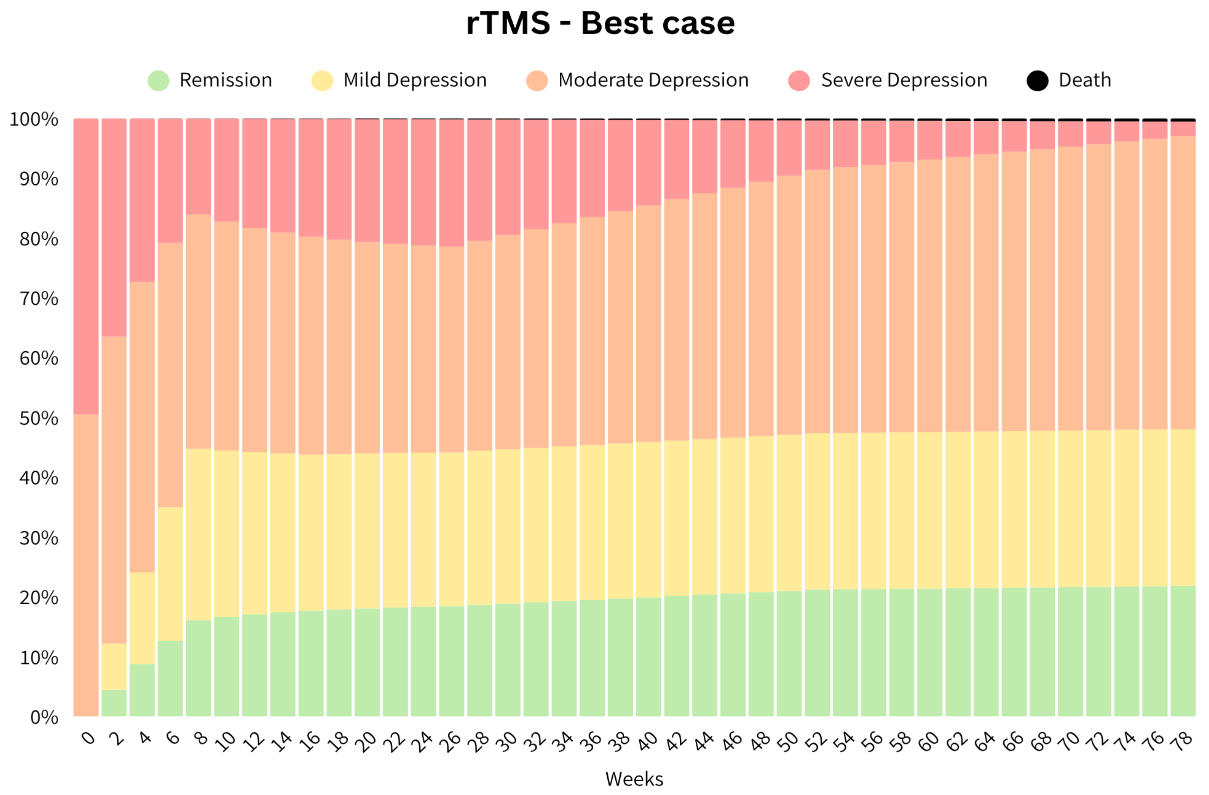
rTMS - Best case
Remission
Mild Depression
Moderate Depression
Severe Depression
Death
100%
90%
80%
70%
60%
50%
40%
30%
20%
10%
0%
0 2 4 6 8 10 12 14 16 18 20 22 24 26 28 30 32 34 36 38 40 42 44 46 48 50 52 54 56 58 60 62 64 66 68 70 72 74 76 78
Weeks

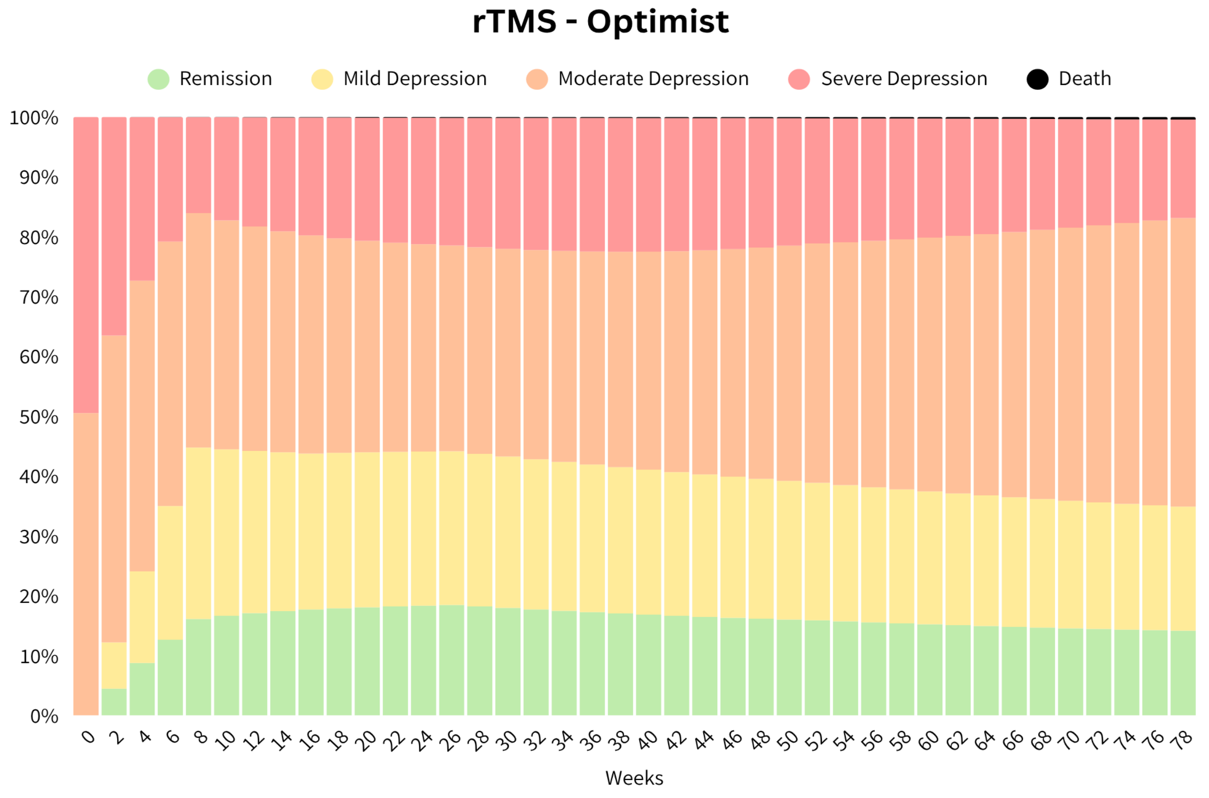
rTMS - Optimist
Remission
Mild Depression
Moderate Depression
Severe Depression
Death
100%
90%
80%
70%
60%
50%
40%
30%
20%
10%
0%
0 2 4 6 8 10 12 14 16 18 20 22 24 26 28 30 32 34 36 38 40 42 44 46 48 50 52 54 56 58 60 62 64 66 68 70 72 74 76 78
Weeks

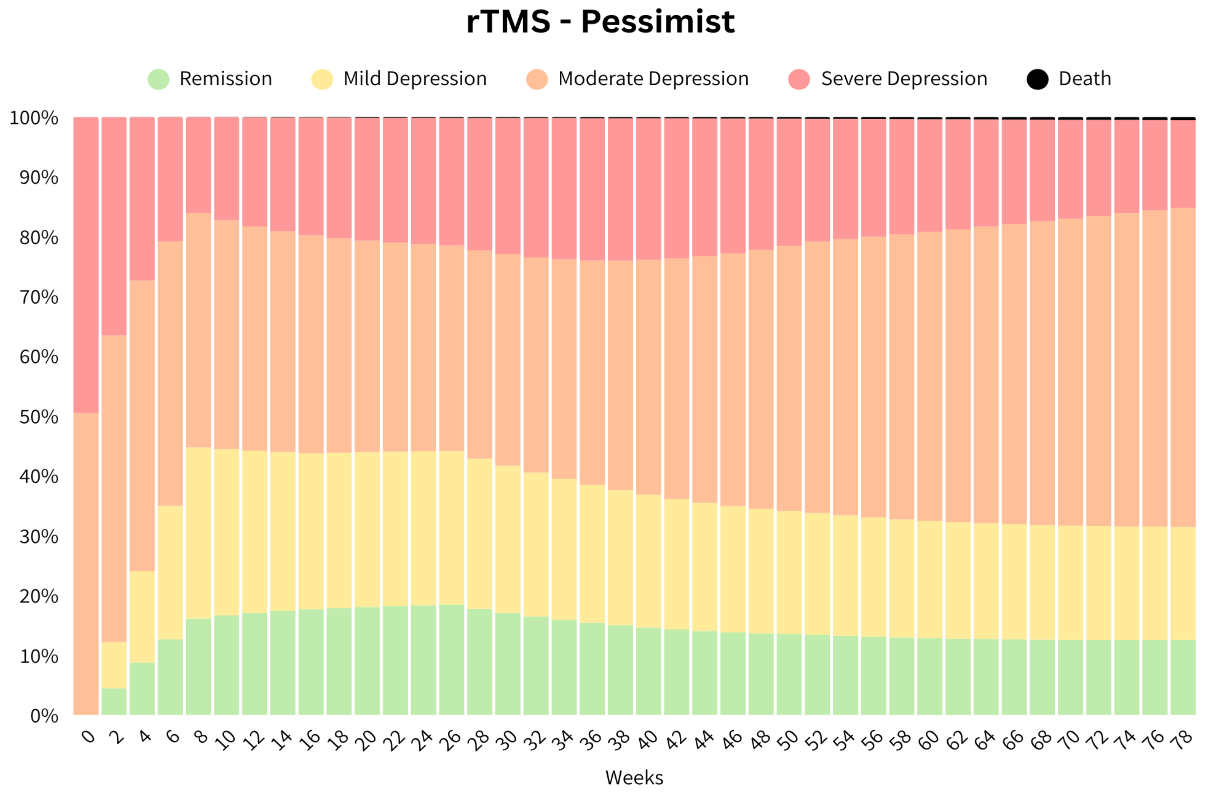
rTMS - Pessimist
Remission
Mild Depression
Moderate Depression
Severe Depression
Death
100%
90%
80%
70%
60%
50%
40%
30%
20%
10%
0%
0 2 4 6 8 10 12 14 16 18 20 22 24 26 28 30 32 34 36 38 40 42 44 46 48 50 52 54 56 58 60 62 64 66 68 70 72 74 76 78
Weeks

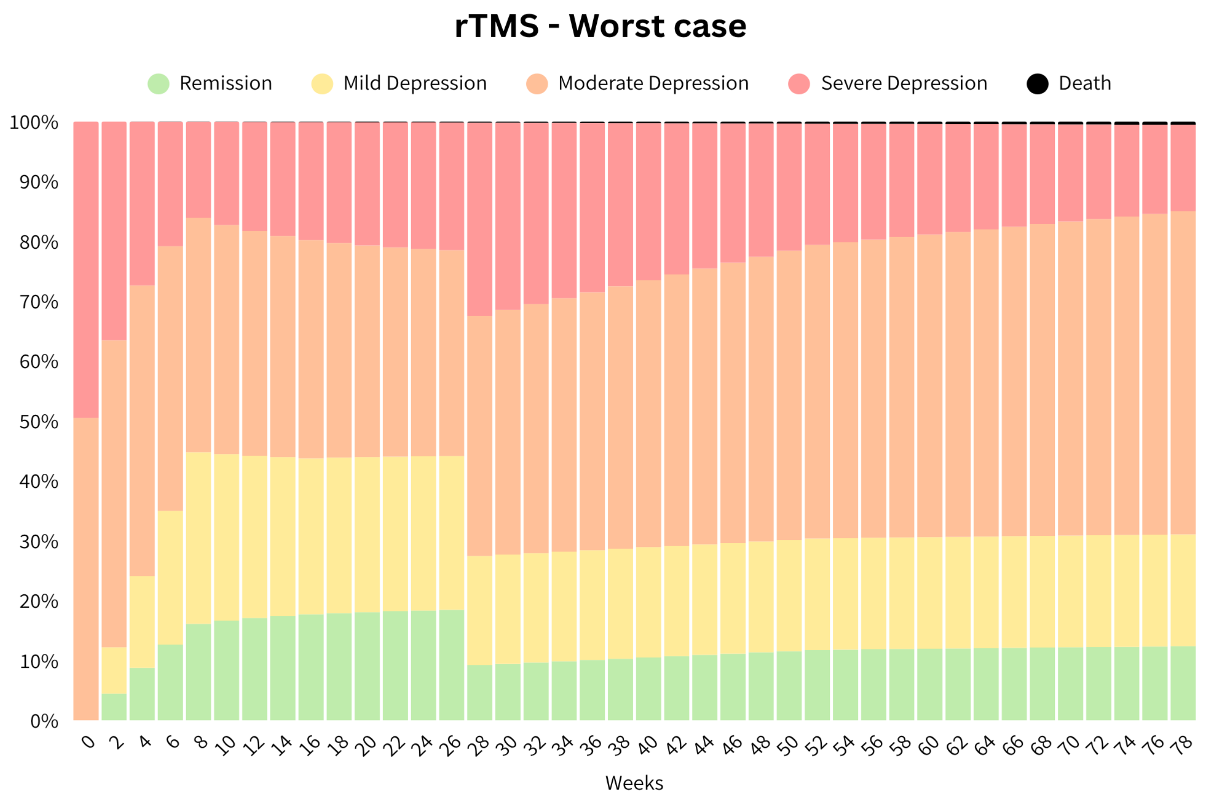
rTMS - Worst case
Remission
Mild Depression
Moderate Depression
Severe Depression
Death
100%
90%
80%
70%
60%
50%
40%
30%
20%
10%
0%
0
2
4
6
8
10
12
14
16
18
20
22
24
26
28
30
32
34
36
38
40
42
44
46
48
50
52
54
56
58
60
62
64
66
68
70
72
74
76
78
Weeks

3.10 Scenario analyses

Table S30: Cost-effectiveness results for all modelled scenario analyses

Fully Incremental Scenario Analyses

Base case findings

NHS & PSS Perspective

	Costs	QALYS	Incremental Costs	Incremental QALYs	ICER
**TAU**	£5,940	0.9253			
**rTMS**	£6,521	0.9734	£581	0.0480	£12,093
**iTBS**	£6,709	0.9847	£189	0.0113	£16,621

Broader societal perspective

	Costs	QALYS	Informal care hours	Incremental Costs	Incremental Informal care hours	Incremental QALYs	ICER
**rTMS**	£9,861	0.9734	217				
**iTBS**	£9,864	0.9847	211	£3	-5.7	0.0113	£269
**TAU**	£9,880	0.9253	240	£16	28.4	-0.0594	Dominated

Extrapolation - Best case

NHS & PSS Perspective

	Costs	QALYS	Incremental Costs	Incremental QALYs	ICER
**TAU**	£5,736	0.9201			
**rTMS**	£6,044	0.9973	£308	0.0772	£3,985
**iTBS**	£6,217	1.0136	£173	0.0163	£10,570

Broader societal perspective

	Costs	QALYS	Informal care hours	Incremental Costs	Incremental Informal care hours	Incremental QALYs	ICER
**iTBS**	£8,962	1.0136	194.9				
**rTMS**	£9,058	0.9973	203.4	£96	8.5	-0.0163	Dominated
**TAU**	£9,689	0.9201	239.5	£727	44.6	-0.0935	Dominated

Extrapolation - Worst case

NHS & PSS Perspective

	Costs	QALYS	Incremental		
			Costs	QALYs	ICER
**TAU**	£5,908	0.9173			
**rTMS**	£6,651	0.9503	£743	0.0329	£22,568
**iTBS**	£6,866	0.9565	£215	0.0063	£34,242

Broader societal perspective

	Costs	QALYS	Informal care hours	Incremental			
				Costs	Informal care hours	QALYs	ICER
**TAU**	£9,858	0.9173	240.3				
**rTMS**	£10,195	0.9503	225.7	£336	-14.6	0.0329	£10,219
**iTBS**	£10,309	0.9565	222.7	£115	-3.0	0.0063	£18,263

Extrapolation - Optimist

NHS & PSS Perspective

	Costs	QALYS	Incremental		
			Costs	QALYs	ICER
**TAU**	£5,951	0.9258			
**rTMS**	£6,521	0.9767	£570	0.0509	£11,190
**iTBS**	£6,704	0.9892	£183	0.0125	£14,628

Broader societal perspective

	Costs	QALYS	Informal care hours	Incremental			
				Costs	Informal care hours	QALYs	ICER
**iTBS**	£9,807	0.9892	208.6				
**rTMS**	£9,829	0.9767	215.0	£22	6.4	-0.0125	Dominated
**TAU**	£9,896	0.9258	239.2	£90	30.6	-0.0634	Dominated

Extrapolation - Pessimist

NHS & PSS Perspective

	Costs	QALYS	Incremental		
			Costs	QALYs	ICER
**TAU**	£5,674	0.9233			
**rTMS**	£6,315	0.9671	£641	0.0438	£14,628

**iTBS**	£6,515	0.9768	£200	0.0096	£20,783

Broader societal perspective

	Costs	QALYS	Informal care hours	Incremental			
				Costs	Informal care hours	QALYs	ICER
**TAU**	£9,619	0.9233	241.0				
**rTMS**	£9,717	0.9671	220.7	£98	-20.3	0.0438	£2,229
**iTBS**	£9,760	0.9768	215.9	£43	-4.8	0.0096	£4,528

No death state

NHS & PSS Perspective

	Costs	QALYS	Incremental		
			Costs	QALYs	ICER
**TAU**	£5,867	0.9234			
**rTMS**	£6,461	0.9712	£594	0.0478	£12,444
**iTBS**	£6,649	0.9826	£188	0.0114	£16,483

Broader societal perspective

	Costs	QALYS	Informal care hours	Incremental			
				Costs	Informal care hours	QALYs	ICER
**iTBS**	£9,821	0.9712	217.8				
**rTMS**	£9,822	0.9826	212.1	£2	-5.7	0.0114	£134
**TAU**	£9,830	0.9234	240.6	£7	28.6	-0.0591	Dominated

SMD trial population and inputs

NHS & PSS Perspective

	Costs	QALYS	Incremental		
			Costs	QALYs	ICER
**TAU**	£5,864	0.6148			
**rTMS**	£6,468	0.7012	£604	0.0865	£6,990
**iTBS**	£6,656	0.7217	£188	0.0205	£9,172

Broader societal perspective

	Costs	QALYS	Informal care hours	Incremental			
				Costs	Informal care hours	QALYs	ICER

**TAU**	£9,813	0.6148	238.7				
**rTMS**	£9,816	0.7012	216.0	£3	-22.6	0.0865	£33
**iTBS**	£9,819	0.7217	210.4	£3	-5.7	0.0205	£157

BRIGHTMIND trial population and inputs

NHS & PSS Perspective

	Costs	QALYS	Incremental Costs	Incremental QALYs	ICER
**TAU**	£4,087	0.9268			
**rTMS**	£4,896	0.9721	£809	0.0453	Ext Dominated
**iTBS**	£5,041	0.9834	£953	0.0566	£16,842

Broader societal perspective

	Costs	QALYS	Informal care hours	Incremental Costs	Incremental Informal care hours	Incremental QALYs	ICER
**TAU**	£8,031	0.9268	239.5				
**iTBS**	£8,231	0.9834	212.2	£200	-27.3	0.0566	£3,533
**rTMS**	£8,269	0.9721	217.9	£38	5.7	-0.0113	Dominated

TAU definition - Specialist service

NHS & PSS Perspective

	Costs	QALYS	Incremental Costs	Incremental QALYs	ICER
**TAU**	£5,567	0.9494			
**rTMS**	£6,320	0.9851	£752	0.0358	Ext Dominated
**iTBS**	£6,511	0.9965	£944	0.0471	£20,049

Broader societal perspective

	Costs	QALYS	Informal care hours	Incremental Costs	Incremental Informal care hours	Incremental QALYs	ICER
**TAU**	£9,193	0.9494	224.5				
**rTMS**	£9,471	0.9851	209.0	£278	-15.5	0.0358	Ext Dominated
**iTBS**	£9,477	0.9965	203.3	£284	-21.2	0.0471	£6,036

BRIGHTMIND TMS delivery cost

NHS & PSS Perspective

	Costs	QALYS	Incremental Costs	Incremental QALYs	ICER
**TAU**	£5,823	0.9204			
**rTMS**	£6,674	0.9799	£851	0.0595	£14,304
**iTBS**	£6,786	0.9685	£113	-0.0114	Dominated

Broader societal perspective

	Costs	QALYS	Informal care hours	Incremental			
				Costs	Informal care hours	QALYs	ICER
**TAU**	£9,778	0.9204	240.1				
**iTBS**	£9,841	0.9799	211.7	£63	-28.4	0.0595	£1,067
**rTMS**	£10,140	0.9685	217.5	£299	5.8	-0.0114	Dominated

TAU definition - Specialist service

NHS & PSS Perspective

	Costs	QALYS	Incremental Costs	Incremental QALYs	ICER
**TAU**	£5,567	0.9494			
**rTMS**	£6,320	0.9851	£752	0.0358	Ext Dominated
**iTBS**	£6,511	0.9965	£944	0.0471	£20,049

Broader societal perspective

	Costs	QALYS	Informal care hours	Incremental			
				Costs	Informal care hours	QALYs	ICER
**TAU**	£9,193	0.9494	224.5				
**rTMS**	£9,471	0.9851	209.0	£278	-15.5	0.0358	Ext Dominated
**iTBS**	£9,477	0.9965	203.3	£284	-21.2	0.0471	£6,036

3.11 Operational sensitivity analysis

Table S31: Operational sensitivity analysis: M=Minutes; S=Sessions; T=Throughput

Intervention profiles			Incremental net health benefit				Probability of being cost-effective alternative														ICER vs TAU	
			$\lambda =$ £20,000		$\lambda =$ £30,000		Three-way comparison						Two-way comparison									
							$\lambda =$ £20,000			$\lambda =$ £30,000			$\lambda =$ £20,000				$\lambda =$ £30,000					
M	S	T	iTBS	rTMS	iTBS	rTMS	iTBS	rTMS	TAU	iTBS	rTMS	TAU	iTBS	TAU	rTMS	TAU	iTBS	TAU	rTMS	TAU	iTBS	rTMS
15	18	10	−0.01	−0.02	0.01	0.01	0.17	0.06	0.76	0.48	0.08	0.44	0.23	0.77	0.21	0.80	0.54	0.46	0.45	0.55	£24,729	£26,644
30	18	10	−0.03	−0.03	0.00	0.00	0.13	0.04	0.83	0.33	0.05	0.62	0.16	0.84	0.15	0.85	0.37	0.63	0.30	0.70	£29,047	£31,983
45	18	10	−0.04	−0.04	−0.01	−0.01	0.10	0.03	0.87	0.23	0.03	0.74	0.12	0.88	0.11	0.89	0.26	0.74	0.20	0.80	£33,366	£37,322
60	18	10	−0.05	−0.05	−0.02	−0.02	0.08	0.02	0.90	0.16	0.02	0.81	0.10	0.90	0.08	0.92	0.18	0.82	0.14	0.86	£37,685	£42,661
15	22	10	−0.02	−0.02	0.01	0.00	0.16	0.06	0.78	0.43	0.07	0.49	0.21	0.79	0.19	0.81	0.49	0.51	0.40	0.60	£25,820	£27,994
30	22	10	−0.03	−0.03	0.00	−0.01	0.11	0.04	0.85	0.27	0.04	0.68	0.14	0.86	0.13	0.87	0.31	0.69	0.25	0.75	£31,099	£34,519
45	22	10	−0.05	−0.05	−0.01	−0.02	0.08	0.02	0.89	0.18	0.03	0.79	0.10	0.90	0.09	0.91	0.21	0.79	0.16	0.84	£36,377	£41,045
60	22	10	−0.06	−0.07	−0.02	−0.03	0.06	0.02	0.92	0.12	0.02	0.86	0.07	0.93	0.06	0.94	0.14	0.86	0.11	0.89	£41,656	£47,570
15	26	10	−0.02	−0.02	0.01	0.00	0.15	0.05	0.80	0.40	0.07	0.54	0.19	0.81	0.17	0.83	0.45	0.55	0.36	0.64	£26,912	£29,343
30	26	10	−0.04	−0.04	−0.01	−0.01	0.10	0.03	0.87	0.23	0.03	0.73	0.12	0.88	0.11	0.89	0.26	0.74	0.20	0.80	£33,150	£37,055
45	26	10	−0.06	−0.06	−0.02	−0.02	0.07	0.02	0.91	0.15	0.02	0.83	0.09	0.91	0.07	0.93	0.16	0.84	0.13	0.87	£39,389	£44,767
60	26	10	−0.08	−0.08	−0.03	−0.04	0.05	0.01	0.94	0.10	0.01	0.89	0.06	0.94	0.05	0.95	0.11	0.89	0.08	0.92	£45,627	£52,479
15	30	10	−0.02	−0.03	0.00	0.00	0.14	0.05	0.82	0.36	0.06	0.59	0.18	0.82	0.16	0.84	0.41	0.59	0.32	0.68	£28,004	£30,693
30	30	10	−0.05	−0.05	−0.01	−0.02	0.09	0.03	0.89	0.20	0.03	0.77	0.11	0.89	0.10	0.90	0.22	0.78	0.17	0.83	£35,202	£39,591
45	30	10	−0.07	−0.07	−0.02	−0.03	0.06	0.02	0.93	0.12	0.02	0.87	0.07	0.93	0.06	0.94	0.13	0.87	0.10	0.90	£42,400	£48,490
60	30	10	−0.09	−0.09	−0.04	−0.04	0.04	0.01	0.95	0.08	0.01	0.91	0.05	0.95	0.04	0.96	0.09	0.91	0.07	0.93	£49,598	£57,388
15	18	25	0.03	0.03	0.04	0.04	0.51	0.39	0.09	0.80	0.18	0.01	0.88	0.12	0.88	0.12	0.98	0.02	0.98	0.02	£8,661	£6,781
30	18	25	0.02	0.02	0.03	0.03	0.41	0.26	0.33	0.79	0.17	0.03	0.63	0.37	0.63	0.37	0.96	0.04	0.95	0.05	£12,980	£12,120
45	18	25	0.01	0.01	0.03	0.02	0.30	0.15	0.55	0.75	0.16	0.09	0.42	0.58	0.41	0.59	0.90	0.10	0.85	0.15	£17,299	£17,459
60	18	25	0.00	−0.01	0.02	0.01	0.22	0.09	0.70	0.61	0.12	0.27	0.29	0.71	0.27	0.73	0.71	0.29	0.61	0.39	£21,618	£22,798
15	22	25	0.03	0.03	0.04	0.04	0.49	0.37	0.14	0.80	0.18	0.02	0.83	0.17	0.84	0.16	0.98	0.02	0.97	0.03	£9,753	£8,131
30	22	25	0.01	0.01	0.03	0.02	0.36	0.20	0.45	0.78	0.17	0.05	0.52	0.48	0.51	0.49	0.94	0.06	0.91	0.09	£15,032	£14,656
45	22	25	0.00	0.00	0.02	0.01	0.24	0.10	0.66	0.67	0.13	0.20	0.32	0.68	0.31	0.69	0.79	0.21	0.69	0.31	£20,310	£21,182
60	22	25	−0.02	−0.02	0.01	0.00	0.16	0.06	0.78	0.44	0.07	0.49	0.21	0.79	0.19	0.81	0.50	0.50	0.41	0.59	£25,589	£27,707
15	26	25	0.03	0.03	0.04	0.03	0.47	0.34	0.19	0.80	0.18	0.02	0.77	0.23	0.78	0.22	0.97	0.03	0.97	0.03	£10,845	£9,480
30	26	25	0.01	0.01	0.03	0.02	0.30	0.15	0.55	0.75	0.16	0.09	0.43	0.57	0.42	0.58	0.90	0.10	0.85	0.15	£17,083	£17,192
45	26	25	−0.01	−0.01	0.01	0.01	0.19	0.07	0.74	0.54	0.10	0.36	0.25	0.75	0.24	0.77	0.62	0.38	0.52	0.48	£23,321	£24,904
60	26	25	−0.03	−0.03	0.00	0.00	0.12	0.04	0.84	0.31	0.05	0.64	0.16	0.84	0.14	0.86	0.36	0.64	0.28	0.72	£29,560	£32,616
15	30	25	0.02	0.02	0.04	0.03	0.44	0.30	0.25	0.80	0.18	0.03	0.70	0.30	0.71	0.29	0.97	0.03	0.96	0.04	£11,937	£10,830

Intervention profiles			Incremental net health benefit				Probability of being cost-effective alternative														ICER vs TAU	
			$\lambda =$ £20,000		$\lambda =$ £30,000		Three-way comparison						Two-way comparison									
							$\lambda =$ £20,000			$\lambda =$ £30,000			$\lambda =$ £20,000				$\lambda =$ £30,000					
30	30	25	0.00	0.00	0.02	0.02	0.26	0.12	0.62	0.71	0.14	0.15	0.36	0.64	0.34	0.66	0.84	0.16	0.76	0.24	£19,135	£19,728
45	30	25	−0.02	−0.02	0.01	0.00	0.15	0.06	0.79	0.42	0.07	0.52	0.20	0.80	0.18	0.82	0.47	0.53	0.38	0.62	£26,333	£28,627
60	30	25	−0.04	−0.04	−0.01	−0.01	0.10	0.03	0.88	0.23	0.03	0.74	0.12	0.88	0.11	0.89	0.26	0.74	0.20	0.80	£33,531	£37,525
15	18	40	0.05	0.04	0.05	0.05	0.54	0.43	0.03	0.81	0.19	0.01	0.96	0.04	0.96	0.04	0.99	0.01	0.99	0.01	£4,644	£1,815
30	18	40	0.03	0.03	0.04	0.04	0.51	0.39	0.10	0.80	0.18	0.02	0.86	0.14	0.87	0.13	0.98	0.02	0.98	0.02	£8,963	£7,154
45	18	40	0.02	0.02	0.03	0.03	0.41	0.25	0.34	0.79	0.17	0.03	0.61	0.39	0.62	0.38	0.96	0.04	0.95	0.05	£13,282	£12,493
60	18	40	0.01	0.01	0.02	0.02	0.29	0.15	0.57	0.75	0.16	0.10	0.41	0.59	0.40	0.60	0.89	0.11	0.83	0.17	£17,601	£17,832
15	22	40	0.04	0.04	0.05	0.04	0.53	0.43	0.04	0.81	0.18	0.01	0.95	0.05	0.95	0.05	0.99	0.01	0.99	0.01	£5,736	£3,165
30	22	40	0.03	0.02	0.04	0.03	0.47	0.33	0.20	0.80	0.18	0.02	0.75	0.25	0.77	0.23	0.97	0.03	0.97	0.03	£11,015	£9,691
45	22	40	0.01	0.01	0.03	0.02	0.32	0.17	0.51	0.77	0.16	0.07	0.46	0.54	0.45	0.55	0.93	0.07	0.88	0.12	£16,293	£16,216
60	22	40	0.00	−0.01	0.02	0.01	0.22	0.09	0.69	0.61	0.12	0.27	0.29	0.71	0.27	0.73	0.72	0.28	0.61	0.39	£21,572	£22,741
15	26	40	0.04	0.04	0.05	0.04	0.53	0.42	0.06	0.81	0.18	0.01	0.93	0.07	0.93	0.07	0.99	0.01	0.99	0.01	£6,828	£4,515
30	26	40	0.02	0.02	0.03	0.03	0.41	0.26	0.33	0.79	0.17	0.03	0.63	0.37	0.63	0.37	0.96	0.04	0.95	0.05	£13,066	£12,227
45	26	40	0.00	0.00	0.02	0.02	0.26	0.12	0.62	0.70	0.14	0.15	0.36	0.64	0.34	0.66	0.83	0.17	0.75	0.25	£19,305	£19,939
60	26	40	−0.02	−0.02	0.01	0.00	0.16	0.06	0.78	0.44	0.07	0.48	0.21	0.79	0.19	0.81	0.50	0.50	0.41	0.59	£25,543	£27,650
15	30	40	0.04	0.03	0.04	0.04	0.52	0.41	0.08	0.80	0.18	0.01	0.90	0.10	0.91	0.09	0.98	0.02	0.98	0.02	£7,920	£5,864
30	30	40	0.01	0.01	0.03	0.02	0.35	0.20	0.45	0.78	0.17	0.05	0.52	0.48	0.51	0.49	0.94	0.06	0.91	0.09	£15,118	£14,763
45	30	40	−0.01	−0.01	0.02	0.01	0.20	0.08	0.71	0.58	0.11	0.31	0.27	0.73	0.26	0.74	0.68	0.32	0.57	0.43	£22,316	£23,661
60	30	40	−0.03	−0.03	0.00	0.00	0.13	0.04	0.84	0.31	0.05	0.64	0.16	0.84	0.14	0.86	0.36	0.64	0.28	0.72	£29,514	£32,559
15	18	55	0.05	0.05	0.05	0.05	0.54	0.44	0.02	0.81	0.19	0.01	0.97	0.03	0.97	0.03	0.99	0.01	0.99	0.01	£2,819	−£442
30	18	55	0.04	0.04	0.05	0.04	0.52	0.42	0.06	0.80	0.18	0.01	0.92	0.08	0.93	0.07	0.99	0.02	0.98	0.02	£7,137	£4,897
45	18	55	0.03	0.02	0.04	0.03	0.46	0.32	0.23	0.80	0.18	0.02	0.73	0.27	0.73	0.27	0.97	0.03	0.96	0.04	£11,456	£10,236
60	18	55	0.01	0.01	0.03	0.02	0.34	0.18	0.48	0.77	0.17	0.06	0.49	0.51	0.48	0.52	0.93	0.07	0.90	0.10	£15,775	£15,575
15	22	55	0.05	0.05	0.05	0.05	0.54	0.44	0.02	0.81	0.19	0.01	0.97	0.03	0.97	0.03	0.99	0.01	0.99	0.01	£3,910	£908
30	22	55	0.03	0.03	0.04	0.04	0.50	0.38	0.11	0.80	0.18	0.02	0.85	0.15	0.86	0.14	0.98	0.02	0.98	0.02	£9,189	£7,433
45	22	55	0.02	0.01	0.03	0.03	0.37	0.22	0.41	0.79	0.17	0.04	0.55	0.45	0.55	0.45	0.95	0.05	0.92	0.08	£14,468	£13,959
60	22	55	0.00	0.00	0.02	0.02	0.25	0.11	0.64	0.69	0.14	0.17	0.34	0.66	0.32	0.68	0.81	0.19	0.72	0.28	£19,746	£20,484
15	26	55	0.04	0.04	0.05	0.04	0.53	0.43	0.03	0.81	0.19	0.01	0.96	0.04	0.96	0.04	0.99	0.01	0.99	0.01	£5,002	£2,258
30	26	55	0.03	0.02	0.04	0.03	0.46	0.32	0.21	0.80	0.18	0.02	0.74	0.26	0.75	0.25	0.97	0.03	0.96	0.04	£11,240	£9,969
45	26	55	0.01	0.01	0.02	0.02	0.29	0.15	0.56	0.75	0.16	0.10	0.42	0.58	0.40	0.60	0.89	0.11	0.84	0.16	£17,479	£17,681
60	26	55	−0.01	−0.01	0.01	0.01	0.19	0.07	0.74	0.52	0.10	0.39	0.25	0.75	0.23	0.77	0.60	0.41	0.50	0.50	£23,717	£25,393
15	30	55	0.04	0.04	0.05	0.04	0.53	0.42	0.05	0.81	0.18	0.01	0.94	0.06	0.94	0.06	0.99	0.01	0.99	0.01	£6,094	£3,607
30	30	55	0.02	0.02	0.03	0.03	0.41	0.25	0.34	0.79	0.17	0.03	0.61	0.39	0.62	0.38	0.96	0.04	0.95	0.05	£13,292	£12,506
45	30	55	0.00	0.00	0.02	0.01	0.23	0.10	0.67	0.66	0.13	0.21	0.32	0.68	0.30	0.70	0.78	0.22	0.68	0.32	£20,490	£21,404
60	30	55	−0.02	−0.02	0.00	0.00	0.14	0.05	0.81	0.37	0.06	0.58	0.18	0.82	0.16	0.84	0.42	0.58	0.33	0.67	£27,688	£30,302

Intervention profiles			Incremental net health benefit λ = £20,000		λ = £30,000		Probability of being cost-effective alternative: Three-way comparison λ = £20,000			λ = £30,000			Two-way comparison λ = £20,000				λ = £30,000				ICER vs TAU	
15	18	70	0.05	0.05	0.06	0.05	0.54	0.44	0.02	0.81	0.19	0.01	0.98	0.02	0.98	0.02	0.99	0.01	0.99	0.01	£1,775	−£1,732
30	18	70	0.04	0.04	0.05	0.04	0.53	0.42	0.05	0.81	0.18	0.01	0.94	0.06	0.94	0.06	0.99	0.01	0.99	0.01	£6,094	£3,607
45	18	70	0.03	0.03	0.04	0.03	0.48	0.35	0.17	0.80	0.18	0.02	0.79	0.21	0.80	0.20	0.98	0.02	0.97	0.03	£10,413	£8,947
60	18	70	0.02	0.01	0.03	0.03	0.37	0.21	0.43	0.78	0.17	0.05	0.54	0.46	0.53	0.47	0.95	0.05	0.92	0.08	£14,732	£14,286
15	22	70	0.05	0.05	0.05	0.05	0.54	0.44	0.02	0.81	0.19	0.01	0.97	0.03	0.97	0.03	0.99	0.01	0.99	0.01	£2,867	−£382
30	22	70	0.04	0.03	0.04	0.04	0.52	0.40	0.08	0.80	0.18	0.01	0.89	0.11	0.90	0.10	0.98	0.02	0.98	0.02	£8,146	£6,144
45	22	70	0.02	0.02	0.03	0.03	0.40	0.25	0.35	0.79	0.17	0.04	0.61	0.39	0.61	0.39	0.96	0.04	0.94	0.06	£13,424	£12,669
60	22	70	0.00	0.00	0.02	0.02	0.27	0.13	0.61	0.72	0.15	0.13	0.37	0.63	0.36	0.65	0.86	0.14	0.78	0.22	£18,703	£19,195
15	26	70	0.05	0.05	0.05	0.05	0.54	0.44	0.03	0.81	0.19	0.01	0.97	0.03	0.97	0.03	0.99	0.01	0.99	0.01	£3,959	£968
30	26	70	0.03	0.03	0.04	0.03	0.48	0.36	0.16	0.80	0.18	0.02	0.80	0.20	0.81	0.19	0.98	0.02	0.97	0.03	£10,197	£8,680
45	26	70	0.01	0.01	0.03	0.02	0.32	0.16	0.52	0.77	0.16	0.07	0.46	0.54	0.44	0.56	0.92	0.08	0.88	0.12	£16,435	£16,392
60	26	70	−0.01	−0.01	0.01	0.01	0.20	0.08	0.72	0.56	0.11	0.33	0.27	0.73	0.25	0.75	0.65	0.35	0.56	0.44	£22,674	£24,104
15	30	70	0.04	0.04	0.05	0.04	0.53	0.43	0.03	0.81	0.19	0.01	0.95	0.05	0.96	0.04	0.99	0.01	0.99	0.01	£5,051	£2,317
30	30	70	0.02	0.02	0.04	0.03	0.43	0.29	0.28	0.80	0.18	0.03	0.68	0.32	0.69	0.31	0.97	0.03	0.96	0.05	£12,249	£11,216
45	30	70	0.00	0.00	0.02	0.02	0.25	0.12	0.63	0.70	0.14	0.16	0.35	0.65	0.33	0.67	0.83	0.17	0.73	0.27	£19,447	£20,114
60	30	70	−0.02	−0.02	0.01	0.00	0.15	0.05	0.80	0.40	0.07	0.53	0.20	0.80	0.18	0.82	0.46	0.54	0.37	0.63	£26,645	£29,013
15	18	85	0.06	0.05	0.06	0.05	0.54	0.44	0.01	0.81	0.19	0.00	0.98	0.02	0.98	0.02	0.99	0.01	0.99	0.01	£1,100	−£2,566
30	18	85	0.04	0.04	0.05	0.04	0.53	0.43	0.04	0.81	0.19	0.01	0.95	0.05	0.95	0.05	0.99	0.01	0.99	0.01	£5,419	£2,773
45	18	85	0.03	0.03	0.04	0.04	0.49	0.37	0.13	0.80	0.18	0.02	0.83	0.17	0.84	0.16	0.98	0.02	0.97	0.03	£9,738	£8,112
60	18	85	0.02	0.02	0.03	0.03	0.38	0.23	0.39	0.79	0.17	0.04	0.57	0.43	0.57	0.43	0.96	0.05	0.93	0.07	£14,057	£13,451
15	22	85	0.05	0.05	0.06	0.05	0.54	0.44	0.02	0.81	0.19	0.01	0.97	0.03	0.98	0.02	0.99	0.01	0.99	0.01	£2,192	−£1,216
30	22	85	0.04	0.04	0.04	0.04	0.52	0.41	0.07	0.80	0.18	0.01	0.91	0.09	0.92	0.08	0.98	0.02	0.98	0.02	£7,471	£5,309
45	22	85	0.02	0.02	0.03	0.03	0.42	0.27	0.31	0.80	0.17	0.03	0.65	0.35	0.65	0.35	0.96	0.04	0.95	0.05	£12,749	£11,834
60	22	85	0.01	0.00	0.02	0.02	0.28	0.14	0.58	0.74	0.15	0.11	0.40	0.60	0.38	0.62	0.88	0.12	0.81	0.19	£18,028	£18,360
15	26	85	0.05	0.05	0.05	0.05	0.54	0.44	0.02	0.81	0.19	0.01	0.97	0.03	0.97	0.03	0.99	0.01	0.99	0.01	£3,284	£133
30	26	85	0.03	0.03	0.04	0.04	0.50	0.38	0.13	0.80	0.18	0.02	0.84	0.16	0.85	0.15	0.98	0.02	0.98	0.02	£9,522	£7,845
45	26	85	0.01	0.01	0.03	0.02	0.34	0.18	0.48	0.77	0.17	0.06	0.49	0.51	0.48	0.52	0.93	0.07	0.90	0.10	£15,760	£15,557
60	26	85	−0.01	−0.01	0.02	0.01	0.21	0.09	0.71	0.59	0.11	0.29	0.28	0.72	0.26	0.74	0.69	0.31	0.59	0.41	£21,999	£23,269
15	30	85	0.05	0.04	0.05	0.05	0.54	0.43	0.03	0.81	0.19	0.01	0.96	0.04	0.96	0.04	0.99	0.01	0.99	0.01	£4,375	£1,483
30	30	85	0.03	0.02	0.04	0.03	0.45	0.31	0.24	0.80	0.18	0.03	0.72	0.28	0.73	0.27	0.97	0.03	0.96	0.04	£11,573	£10,381
45	30	85	0.00	0.00	0.02	0.02	0.27	0.12	0.61	0.72	0.15	0.13	0.37	0.63	0.35	0.65	0.86	0.14	0.77	0.23	£18,772	£19,280
60	30	85	−0.02	−0.02	0.01	0.00	0.16	0.06	0.78	0.43	0.07	0.50	0.21	0.79	0.19	0.81	0.49	0.51	0.39	0.61	£25,970	£28,178
15	18	100	0.06	0.06	0.06	0.05	0.54	0.45	0.01	0.81	0.19	0.00	0.98	0.02	0.99	0.01	0.99	0.01	0.99	0.01	£628	−£3,150
30	18	100	0.04	0.04	0.05	0.04	0.53	0.43	0.03	0.81	0.19	0.01	0.96	0.04	0.96	0.04	0.99	0.01	0.99	0.01	£4,946	£2,189
45	18	100	0.03	0.03	0.04	0.04	0.50	0.38	0.12	0.80	0.18	0.02	0.85	0.15	0.86	0.14	0.98	0.02	0.98	0.02	£9,265	£7,528

Intervention profiles			Incremental net health benefit				Probability of being cost-effective alternative																ICER vs TAU	
			$\lambda =$ £20,000		$\lambda =$ £30,000		Three-way comparison						Two-way comparison									
							$\lambda =$ £20,000			$\lambda =$ £30,000			$\lambda =$ £20,000				$\lambda =$ £30,000					
60	18	100	0.02	0.02	0.03	0.03	0.40	0.24	0.36	0.79	0.17	0.04	0.60	0.40	0.60	0.40	0.96	0.04	0.94	0.06	£13,584	£12,867
15	22	100	0.05	0.05	0.06	0.05	0.54	0.44	0.02	0.81	0.19	0.01	0.98	0.02	0.98	0.02	0.99	0.01	0.99	0.01	£1,719	−£1,801
30	22	100	0.04	0.04	0.05	0.04	0.52	0.42	0.06	0.80	0.18	0.01	0.92	0.08	0.93	0.07	0.99	0.01	0.99	0.01	£6,998	£4,725
45	22	100	0.02	0.02	0.04	0.03	0.43	0.29	0.28	0.80	0.18	0.03	0.67	0.33	0.69	0.32	0.97	0.03	0.95	0.05	£12,277	£11,250
60	22	100	0.01	0.01	0.02	0.02	0.29	0.15	0.56	0.75	0.16	0.10	0.41	0.59	0.40	0.60	0.89	0.11	0.84	0.16	£17,555	£17,776
15	26	100	0.05	0.05	0.05	0.05	0.54	0.44	0.02	0.81	0.19	0.01	0.97	0.03	0.97	0.03	0.99	0.01	0.99	0.01	£2,811	−£451
30	26	100	0.03	0.03	0.04	0.04	0.51	0.39	0.11	0.80	0.18	0.02	0.86	0.14	0.87	0.13	0.98	0.02	0.98	0.02	£9,049	£7,261
45	26	100	0.01	0.01	0.03	0.02	0.35	0.19	0.46	0.78	0.17	0.05	0.51	0.49	0.50	0.50	0.94	0.06	0.91	0.09	£15,288	£14,973
60	26	100	0.00	−0.01	0.02	0.01	0.22	0.09	0.69	0.61	0.12	0.27	0.29	0.71	0.28	0.72	0.72	0.28	0.61	0.39	£21,526	£22,685
15	30	100	0.05	0.05	0.05	0.05	0.54	0.44	0.02	0.81	0.19	0.01	0.97	0.03	0.97	0.03	0.99	0.01	0.99	0.01	£3,903	£899
30	30	100	0.03	0.02	0.04	0.03	0.46	0.33	0.20	0.80	0.18	0.02	0.75	0.25	0.76	0.24	0.97	0.03	0.97	0.04	£11,101	£9,797
45	30	100	0.01	0.00	0.02	0.02	0.28	0.13	0.59	0.73	0.15	0.12	0.39	0.61	0.37	0.63	0.87	0.13	0.80	0.20	£18,299	£18,695
60	30	100	−0.02	−0.02	0.01	0.00	0.16	0.06	0.78	0.44	0.08	0.48	0.21	0.79	0.19	0.81	0.51	0.49	0.41	0.59	£25,497	£27,594

Bibliography

[1] R. Morriss, P. M. Briley, L. Webster, M. Abdelghani, S. Barber, P. Bates, C. Brookes, B. Hall, L. Ingram, M. Kurkar, *et al.*, “Connectivity-guided intermittent theta burst versus repetitive transcranial magnetic stimulation for treatment-resistant depression: a randomized controlled trial,” *Nature Medicine*, vol. 30, no. 2, pp. 403–413, 2024.

[2] R. Morriss, A. Garland, N. Nixon, B. Guo, M. James, C. Kaylor-Hughes, R. Moore, R. Ramana, C. Sampson, T. Sweeney, *et al.*, “Efficacy and cost-effectiveness of a specialist depression service versus usual specialist mental health care to manage persistent depression: a randomised controlled trial,” *The Lancet Psychiatry*, vol. 3, no. 9, pp. 821–831, 2016.

[3] M. Hamilton, “A rating scale for depression,” *Journal of neurology, neurosurgery, and psychiatry*, vol. 23, no. 1, p. 56, 1960.

[4] R. Shi, Z. Wang, D. Yang, Y. Hu, Z. Zhang, D. Lan, Y. Su, and Y. Wang, “Short-term and long-term efficacy of accelerated transcranial magnetic stimulation for depression: a systematic review and meta-analysis,” *BMC psychiatry*, vol. 24, no. 1, p. 109, 2024.

[5] A. Qaseem, M. J. Barry, D. Kansagara, and C. G. C. of the American College of Physicians, “Nonpharmacologic versus pharmacologic treatment of adult patients with major depressive disorder: a clinical practice guideline from the american college of physicians,” *Annals of internal medicine*, vol. 164, no. 5, pp. 350–359, 2016.

[6] L. Bojke, M. Soares, K. Claxton, A. Colson, A. Fox, C. Jackson, D. Jankovic, A. Morton, L. Sharples, and A. Taylor, “Developing a reference protocol for structured expert elicitation in health-care decision-making: a mixed-methods study.,” *Health Technology Assessment (Winchester, England)*, vol. 25, no. 37, p. 1, 2021.

[7] L. Bojke, M. Soares, K. Claxton, A. Colson, A. Fox, C. Jackson, D. Jankovic, A. Morton, L. Sharples, and A. Taylor, “Structured expert elicitation resources (STEER).” Available at: https://www.york.ac.uk/che/economic-evaluation/steer/.

[8] Dr Alex O’Neill-Kerr, *Expert Profile*. https://professoralexoneillkerr.co.uk/.

[9] Dr Micheal Kurkar, *Expert Profile*. https://alexmed.co.uk/doctor/dr-micheal-kurkar/.

[10] Dr Mohamed Abdelghani, *Expert Profile*. https://londontmscentre.com/mohamed-abdelghani/.

[11] Dr Mourad Wahba, *Expert Profile*. https://www.ncl.ac.uk/medical-sciences/people/profile/mouradwahba.html.

[12] Ben Baxter, *Expert Profile*. Psychiatric Nurse - NTW Trust.

[13] Dr Richard Barnes, *Clinical Nurse Lead, Physical Treatment Centre, Cumbria, Northumberland, Tyne and Wear NHS Foundation Trust, Hadrian Clinic, Campus for Ageing and Vitality (formerly Newcastle General Hospital)*. https://www.cntw.nhs.uk/.

[14] Dr Sudheer Lankappa, *Expert Profile*. https://institutemh.org.uk/research/centre-for-translational-neuroscience/members-of-the-centre-for-translational-neuroscience/1607-dr-sudheer-lankappa.

[15] D. Ayers, S. Cope, K. Towle, A. Mojebi, T. Marshall, and D. Dhanda, “Structured expert elicitation to inform long-term survival extrapolations using alternative parametric distributions: a case study of car t therapy for relapsed/refractory multiple myeloma,” *BMC Medical Research Methodology*, vol. 22, no. 1, p. 272, 2022.

[16] A. O’Hagan, C. Buck, A. Daneshkhah, J. Eiser, P. Garthwaite, D. Jenkinson, J. Oakley, and T. Rakow, *Uncertain Judgements: Eliciting Experts’ Probabilities*. Chichester, UK: John Wiley & Sons, 2006.

[17] P. H. Garthwaite, J. B. Kadane, and A. O’Hagan, “Statistical methods for eliciting probability distributions,” *Journal of the American Statistical Association*, vol. 100, no. 470, pp. 680–701, 2005.
